# The Well-Being of Companion Animal Caregivers and Their Companion Animals during the COVID-19 Pandemic: Scoping Review

**DOI:** 10.3390/ani13203294

**Published:** 2023-10-22

**Authors:** Samantha K. Brooks, Neil Greenberg

**Affiliations:** Department of Psychological Medicine, King’s College London, Cutcombe Road, London SE5 9RJ, UK

**Keywords:** animal caregivers, companion animals, COVID-19, pet owners, pets, well-being

## Abstract

**Simple Summary:**

Pandemics are predicted to increase in frequency, so it is important that lessons are learned from COVID-19. As half of the world’s population has a companion animal in the home, it is also important to understand the pandemic experiences of both animal caregivers and their animals. Since 2020, a vast amount of literature has been published on the psychological well-being of people with companion animals during the COVID-19 pandemic. This review examined the effect of caring for companion animals on humans’ psychological well-being during this global crisis, as well as the benefits and challenges of having a companion animal during the pandemic and perceived effects on animals’ well-being. We reviewed one hundred and twenty-two studies and found positive, negative, and neutral psychological effects of having a companion animal during the pandemic. Animals were described as providing routine, a sense of purpose, distraction from COVID-19 worries, companionship, and emotional support. However, caregivers also reported worries about access to animal food and veterinary services, fears about COVID-19 transmission, concerns about being unable to financially support their animals, and worries about what would happen to their animals if their caregivers were hospitalized. Animals themselves experienced both positive and negative effects of being in lockdown.

**Abstract:**

It is important to understand the effects of the COVID-19 pandemic on animal caregivers and their companion animals in order to inform responses to future crises. Prior research is inconsistent, with the benefits of animal companionship believed to be overstated. In this scoping review, we searched four electronic databases and hand-searched reference lists of included studies. Over 4000 citations were found, and 122 were included in the review. Reflecting on the pre-COVID literature, quantitative evidence of the association between psychological well-being and animal companionship during the pandemic was mixed, with numerous positive, negative, and null findings reported. Studies highlighted the benefits of animal companionship during the pandemic, with animals reported to provide their caregivers with a routine, a sense of greater purpose, a positive distraction from COVID-19, companionship, and emotional support. However, participants also reported concerns about meeting animals’ needs, fears of animals catching or spreading the virus, and financial worries. Concerns about what would happen to animals if caregivers were hospitalized led some to delay COVID-19 testing or treatment. Animals also experienced benefits (such as increased companionship and calmer mood) and negative impacts (such as increased clinginess and separation anxiety). Companion animals should be a key consideration in emergency preparedness plans.

## 1. Introduction

### 1.1. Well-Being during the COVID-19 Pandemic

In late December 2019, reports began to emerge from Wuhan, China, about a previously unidentified coronavirus (COVID-19) [1]. In March 2020, the World Health Organization declared a global pandemic [2], and countries across the globe went into ‘lockdown’, with restrictions on movement and social contact to control the spread of the virus. Those who tested positive or were symptomatic for COVID-19 or had close contact with infected individuals were instructed to self-isolate; across the world, all individuals (including those without COVID-19 symptoms or contact) were heavily restricted in terms of ability to physically interact with others outside of their own households. Given that humans are social creatures by nature, this raised concerns among scholars about loneliness, isolation, and the potential mental health effects of confinement and lack of social interaction [3]. In February 2020, *The Lancet* published a rapid review by Brooks et al. on the psychological impact of quarantine [4], suggesting that longer length of quarantine, concerns about infection, financial worries, inadequate supplies and information, and the boredom and isolation associated with reduced social contact and inability to leave home could have negative psychological consequences. Potential effects on psychological well-being included post-traumatic stress symptoms, anxiety, distress, irritability, confusion, and anger [4].

Soon after, in April 2020, Hoy-Gerlach et al. [5] published a paper proposing that companion animals (domesticated or domestic-bred animals who live as companions in close contact with humans [6]) may provide benefits to their humans which might address the particular pandemic-related stressors reported by Brooks et al. [4] relating to confinement, boredom, and loneliness. For example, they suggested that seeking physical contact from animals during confinement might reduce anxiety-related physical symptoms in humans, citing the release of oxytocin that occurs during contact with a companion animal [5]. Hoy-Gerlach et al. [5] also suggested that having a dog may be associated with increased physical activity, which could help to ameliorate the anxiety and depression associated with confinement, that the social benefits of companionship from an animal may address the boredom and frustration of confinement; that animals may fill a crucial void in social support during the pandemic; and that companion animals may help their caregivers to maintain daily routines during lockdown. A number of other letters, editorials, and commentaries were also published early in the pandemic, suggesting that companion animals may be beneficial for humans during lockdown. For instance, it was proposed that companion animals could improve both physical and psychological health [7], that animals could buffer against some of the potential drawbacks of remote working [8], that animals could reduce loneliness for older adults [9], that emotional bonds with animals might help caregivers through the uncertainty and stress of the pandemic [10], and that caring for animals could reduce isolation, foster feelings of hope, and provide meaning, comfort, and a sense of routine [11]. At the same time, the media began promoting similar ideas. *The Guardian* in the United Kingdom published ‘*How pets are helping us through the coronavirus crisis*’ [12], while *The Independent* published ‘*Coronavirus: How pets are supporting people through the loneliness of lockdown*’ [13]. Some healthcare provider websites promoted similar information, such as the ‘*How pets help people cope during a pandemic*’ page added to the Sharp HealthCare website [14]. It is possible that these early suggestions and positive media portrayals of the benefits of companion animals, along with lay intuition that people benefit from companion animals [15], contributed to the increased interest in animals and global surge in adoptions and purchases of animals, particularly dogs, early in lockdown [16].

The argument that companion animals could improve the ‘lockdown’ experience for their caregivers during the pandemic appears compelling and of major public health importance due to how many people across the world have companion animals. It is difficult to truly estimate the global prevalence of keeping companion animals, as many countries (particularly in Africa) have not been adequately surveyed [17]. However, there are estimated to be billions of animals kept as companions worldwide, most commonly dogs and cats, with over half of the population in some countries (such as the United States) sharing their homes with companion animals [18]. Emerging markets (including parts of Asia, Sub-Saharan Africa, and Eastern Europe) are also predicted to see an increase in the keeping of companion animals [18]. In other words, if companion animals really could help to mitigate some of the negative psychological effects of living in lockdown, this could potentially affect a substantial proportion of the world’s population. It is therefore important to understand how companion animals might affect the pandemic experience for their caregivers—and, at the same time, recognizing that animals are beings in their own right, it is important to understand how companion animals themselves might be affected by the change in routine caused by a prolonged crisis such as the COVID-19 pandemic. 

### 1.2. Potential Benefits of Companion Animal Caregiving during the Pandemic

There is indeed evidence to support Hoy-Gerlach et al.’s [5] suggestions. Research suggests that physical contact with animals is both comforting and relaxing for humans, with the potential to promote human well-being when physical contact with other people is not possible, such as during the pandemic [19]. Being a companion animal caregiver has been shown to have a moderately significant positive effect on physical activity [20], which in turn positively affects psychological well-being and has been shown to mitigate the negative effect of the COVID-19 pandemic on mental health [21]. Animals have also been shown to be important sources of social support [22], reduce loneliness [23], and play a role in providing routine for their caregivers [24], all of which are likely to be very important during lockdown [4,5]. Social support, in particular, is critical during times of distress [25]; however, social support from other humans during the pandemic was limited due to restrictions on socialization and movement. Given that companion animals are perceived as ‘close others’ and seen as important parts of the family [22], with evidence to suggest that viewing one’s companion animal as part of the family is associated with better well-being [26], it is perhaps understandable that early publications at the start of lockdown suggested that support and companionship from animals might improve their caregivers’ well-being. 

### 1.3. Potential Challenges of Companion Animal Caregiving during the Pandemic

While it may seem intuitive to think that companion animal caregiving fosters psychological wellness in humans [15] and that companionship and social support from animals are likely to be extremely valuable during confinement [5], there may be numerous challenges and risks associated with spending so much more time with companion animals during the pandemic. Hoy-Gerlach et al. [5] note that the logistics and financial strain of caring for animals (which could potentially have been amplified by the pandemic) might exacerbate other pandemic-related stressors, such as worries about finances and COVID-19 transmission risk. Companion animal caregivers also risk ‘caregiver burden’ [27], exacerbated allergy or asthma symptoms [28], and increased risk of injuries (e.g., dog bites) [29]. Additionally, the early days of lockdown saw steep increases in animal adoption [16], which raises concerns about new caregivers and how well they understand and are prepared for the work involved in caring for animals. It is possible that the stresses involved with caring for animals could be magnified during the pandemic due to spending more time at home with animals and having fewer other distractions than usual, which could potentially contribute to the anxiety, frustration, and distress that people may already be feeling [4]. 

### 1.4. Pre-Pandemic Relationship between Companion Animal Caregiving and Human Well-Being

The idea that companion animal caregiving may have both positive and negative effects on caregivers during the COVID-19 pandemic reflects literature on animal caregiving as a whole, i.e., outside of the context of a global crisis. Previous literature is divided and has identified positive, negative, and neutral effects of companion animals on the health and well-being of caregivers, as well as a multitude of benefits and challenges. 

In terms of positive effects, many studies have suggested that companion animals can positively impact the physical and mental health of their caregivers. For example, studies have reported that animals benefit caregivers’ cardiovascular health [30], that people with companion animals have fewer minor health problems such as colds and flu than people without animals [31], and that animal caregivers have fewer sick days than people without companion animals [32]. Reported psychosocial benefits of having a companion animal include reduced loneliness [33], reduced depression [34], and greater positive affect [35]. Dogs, in particular, have been reported to benefit caregivers biologically (e.g., positive effects on blood pressure, heart rate, and cortisol), psychologically (e.g., improved mood and emotion), and socially (e.g., healthy social development in young people and better social relations) [36]. 

However, Herzog [37] suggested that many of the studies supposedly showing positive effects of companion animals on human health could not be replicated and provided evidence that animal caregivers may, in fact, be more at risk than non-caregivers of both psychological and physical ill-health. Indeed, negative health impacts of animal caregiving have also been reported, with studies contradicting those showing positive effects and suggesting animal caregivers have a greater prevalence of asthma, allergic rhinitis, high blood pressure, hypertension, high cholesterol, ulcer, kidney disease, rheumatoid arthritis, migraine, sciatica, depression and panic attacks [38], poorer sleep quality [39], greater anxiety and depression [40], poorer life satisfaction [41] and greater mental health problems overall [42] than people who do not have companion animals. Meanwhile, other studies have found no association at all between companion animals and various health and well-being outcomes such as all-causes mortality [43], physical activity [44], mental health, and loneliness [44]. A number of systematic reviews of the relationship between being a companion animal caregiver and health/well-being have agreed that there is no clear association between the two and that more robust research is needed [24,45,46,47]. The inconsistent findings emerging from the literature may be due to methodological weaknesses such as small samples (typically recruited via convenience sampling) and the tendency to focus on small subgroups [37,40,48]. Additionally, perceptions and experiences of animal caregiving may differ across different populations: for example, it has been reported [49] that older people and people without a life partner report more positive perceptions and experiences of having a companion animal. These differences could also explain the inconsistencies within the literature. 

Despite extant literature reporting very mixed findings of positive, negative, and neutral effects of companion animal caregiving on health and well-being, the media tends to promote unrealistic beliefs about the potentially healing effects of companion animals [15]. The idea that having a companion animal will improve health and happiness has been termed the “pet effect” [15,30], and it is believed to be overstated in the media as it is this effect that garners headlines. In a review of media articles, Herzog [15] found that 70% of articles focused on the positive aspects of companion animal caregiving, emphasizing the health benefits while ignoring the negative or neutral findings.

Overall, extant literature provides a complex picture of the association between animal caregiving and caregiver health. The impact of companion animals on health and well-being during the COVID-19 pandemic is likely to be just as complex. 

### 1.5. Companion Animal Caregiving during Stressful Events

The COVID-19 pandemic is a type of natural disaster [50] and a potentially stressful, traumatic time to live through [4]. Literature relating to companion animal caregiving during stressful events, much like the literature on the relationship between animal caregiving and well-being more generally, provides mixed findings. One study investigating the impact of having a dog on the subjective assessment of critical life events found that dog caregivers assessed stressors to be more stressful than people without dogs [51], although a study of older adults found that animals helped people to cope during times of stress [52]. Literature on animal caregiver well-being during natural disasters is sparse, but Tanaka et al. [53] found that post-traumatic stress disorder was higher in animal caregivers than people without companion animals immediately after an earthquake but lower in caregivers than people without companion animals 4.4 years later; this suggests companion animals may be a stressor in the immediate aftermath of a traumatic event but could be protective during the recovery phase. This may be because looking after animals is another demand for resources and procuring animal food/care products may be more difficult following a disaster. Another study [54] found that after a tornado, there were public health concerns relating to animal caregivers in terms of failure to evacuate because of an animal, attempted re-entry to dangerous sites to save animals, mental health impacts of separation from animals, and refusal to accept medical help until animals were safe. Indeed, a systematic review found that companion animal caregiving impacted disaster-relevant decisions such as evacuation [55]. Additionally, one study reported that dogs were perceived to reduce stress in their caregivers during and after an earthquake, although concern for animals’ safety during the disaster could be a stressor in itself [56]. Taken together, these findings suggest that consideration of companion animals is essential in disaster planning: people may well base their behaviors around their animals. 

### 1.6. Potential Impact of the Pandemic on Companion Animals

It is also important to understand the impact of the pandemic and lockdowns on companion animals themselves rather than reducing them to their potential roles in influencing human well-being. The One Health framework [57], which describes the interconnection between the health of humans, animals, and the environment and is particularly important within the context of COVID-19 [58], suggests that animal and human health are inter-dependent and should be approached collaboratively. 

It might seem intuitive to assume that companion animals could benefit from the pandemic and lockdown restrictions. After all, they would likely be spending more time with their caregivers and presumably have more attention paid to them [59]. However, the change in routine and sudden increase in attention could potentially be stressful and cause behavioral or personality changes in animals (which in turn could also be distressing for their caregivers) [27]. For example, changes in environment and routine have been shown to cause stress in cats, who thrive on routine [60,61]. There may be increases in noise at home, given that more people may be present at any one time and will be spending longer at home; irregular household noises can cause fear, anxiety, and stress in dogs and cats [61,62]. Diminished stimulation could cause boredom in both animals and caregivers [59]. Companion animals such as cats and dogs, who usually spend more time outdoors, may need to be re-trained to the toilet in different locations, which could be confusing for them [59]. Additionally, if animal caregivers are more stressed or anxious during the pandemic themselves [4], this could, in turn, make animals stressed and anxious. For example, evidence suggests that dogs can detect when humans are stressed [63] and that ‘emotional contagion’ between humans and dogs can occur [64]. Stress can lead to both health problems and behavioral problems in animals [65]. Scholars have also raised concerns that animals might experience separation anxiety post-pandemic when their caregivers are no longer able to stay at home all of the time [16] and that unfounded fears that animals might spread COVID-19 could put animals at risk of abuse and even being killed [66]. 

### 1.7. Aims

The aim of this review was to synthesize the large body of literature on caregiver and companion animal well-being during the COVID-19 pandemic to try to establish a clearer picture of the positive and negative impacts and to identify any gaps in the literature. To our knowledge, this is the first review to systematically synthesize the wealth of literature published on companion animal caregiving and psychological well-being during the pandemic. One systematic review was previously conducted [67], which reported both positive and negative implications of the pandemic on both caregivers and animals. However, this review focused on the effects of COVID-19 on caregivers rather than how animals influenced well-being during the pandemic. Additionally, the authors searched only PubMed and Google Scholar, ultimately including only 24 studies. Another review [68] focused on animal caregiving and loneliness both before and during the pandemic, with only six pandemic studies included. Once again, mixed findings were reported, although there appeared to be some evidence that companion animals could reduce loneliness in adult populations. However, a plethora of studies have been published since the searches for the two existing reviews [67,68] were carried out. 

Guided by the One Health framework [57], we aimed to synthesize literature published during the COVID-19 pandemic in order to understand the ways that relationships with companion animals affected the psychological health of humans during this global crisis and the ways in which ‘lockdown’ affected both companion animals and their caregivers. We aimed to use the findings to (a) provide an important contribution to the literature on the relationship between companion animals and caregiver well-being during a unique global crisis and (b) develop recommendations to ensure animal caregivers and their companions are supported in future crises such as pandemics. 

## 2. Materials and Methods

Given that we were aware of a large body of literature on the topic of animal caregiving during the COVID-19 pandemic but that this literature had not previously been synthesized, we opted to carry out a scoping review. This was deemed to be the most appropriate methodology as scoping reviews are recommended for identifying key characteristics of a concept within large bodies of literature and identifying knowledge gaps, potentially as a precursor to a systematic review [69]. This review followed Arksey and O’Malley’s [70] scoping review framework and utilized the PRISMA checklist for scoping reviews [71]. As registration is not deemed necessary for scoping reviews, the review was not pre-registered. The PRISMA-ScR checklist is presented in Supplementary File S1. 

### 2.1. Specifying the Research Question

The first step involved specifying the research question, which was informed by the population, concepts, and context in which we were interested.

The populations of interest in this review were companion animal caregivers and companion animals. Recognizing that companion animals should not be viewed as commodities and are beings in their own right and guided by the principles of the One Health initiative [57], we believed it was important to explore the impact of the pandemic on both human caregivers and their companion animals. Considering the well-being of animals (albeit human-reported), in addition to considering the well-being of humans, allowed us to examine the specific ways in which the pandemic affected animals, whereas we felt that focusing only on human caregivers would reduce animals to their ‘role’ in supporting humans. 

The concepts of interest were, broadly, any ‘well-being-related experiences’. For animal caregivers, this might include mental health disorders (e.g., depression, anxiety) as well as other aspects of psychological well-being, both positive and negative, such as distress, sleep, coping, moods, resilience, and loneliness [4]. For the purposes of this review, we were interested only in these aspects of human well-being in terms of how they relate to companion animals. For companion animals, we were interested in similar outcomes, such as (human-reported) stress and emotions. We were also interested in the perceived benefits and challenges of the pandemic for both caregivers and animals. 

The context of the review was the COVID-19 pandemic and, in particular, how lockdowns and social restrictions affected animal caregivers and their companion animals.

The research question was therefore identified as: *What impact did the COVID-19 pandemic have on the well-being of companion animal caregivers and their companion animals?*

### 2.2. Identifying Relevant Literature

A search strategy was developed to identify literature relevant to answering the research question. The first search string combined COVID-related terminology, such as ‘coronavirus’, ‘lockdown’, and ‘pandemic’. The second search string combined terms relating to companion animals, such as ‘dogs’, ‘cats’, and ‘pets’. The third search string combined well-being-related terms such as ‘psychological’, ‘depression’, and ‘happiness’. The three searches were combined using the Boolean operator ‘AND’. Appendix A presents the full search strategy. 

On 5 August 2023, the first author used this strategy to search four electronic databases (Web of Science, Embase, Medline, and PsycINFO). In order to capture only literature relevant to the COVID-19 pandemic, the searches were limited to studies published after 2019. Searches were also limited to the English language (the language spoken by the authors). To identify other potentially relevant sources, we also searched for reports published by well-known animal charities (e.g., People’s Dispensary for Sick Animals (PDSA), Blue Cross, and Dogs Trust) on their websites and planned to hand-search the reference lists of all studies included in the review.

### 2.3. Selecting Studies

Inclusion and exclusion criteria were defined and are presented in Table 1.

The review was designed to be as broad as possible, and so there were no inclusion criteria relating to study design, measures used, or population size (other than the exception of case studies). 

Studies found by the searches were downloaded to EndNote (X9), where duplicates were removed. Titles and abstracts were then screened for relevance, and any that were clearly not relevant to the review were excluded. Full texts of all remaining citations were located, and the studies were carefully read in their entirety to assess whether they met all inclusion criteria. 

Reference lists of all studies deemed relevant for inclusion in the review were then hand-searched for any additional studies not already found in our original search. New studies that appeared to be relevant based on their titles were added by hand to the EndNote library and underwent the same screening process. 

### 2.4. Charting the Data 

A data extraction form was developed specifically for this study using Microsoft Excel (Version 2309). For each study, the first author systematically extracted data including author names, year of publication, country/countries where the study took place, number of participants, age and gender of (human) participants, species of companion animals, study design, measures relating to human well-being, measures relating to animal well-being, time period of data collection, and key results relating to human well-being, animal well-being, reported challenges of the pandemic and reported positive aspects of the pandemic. 

### 2.5. Collating, Summarizing, and Reporting the Data

Extracted results were imported into NVivo (Version 12) software and analyzed using principles from thematic analysis [72]. 

First, all quantitative data comparing the well-being of animal caregivers with the well-being of people without companion animals were coded ‘Comparisons between caregivers and non-caregivers’. Each of the different well-being outcomes examined were entered into a table alongside columns headed ‘positive findings’ (i.e., data showing companion animals had a significant positive impact on well-being), ‘negative findings’ (i.e., data showing companion animals had a significant negative impact on well-being) and ‘neutral findings’ (i.e., data showing no significant association between having a companion animal and well-being). We went through the quantitative data of each study in turn and classified whether it showed positive, negative, or no associations between companion animals and caregivers’ well-being.

We noted that some studies found significant associations in univariate analyses but not in multivariate analyses. Therefore, secondly, quantitative data, which used multivariate analyses to explore moderators and mediators of the relationship between companion animals and caregivers’ well-being, were coded ‘Factors affecting the association between companion animals and well-being’. We carried out further coding of this data to identify sub-themes. For example, data showing the effect of age or gender on the association between companion animals and well-being were coded as ‘socio-demographic factors’, and data showing the effect of animal species or number of animals in the home were coded as ‘animal-related factors’. 

The next step of our analysis involved synthesizing all of the findings, which were more qualitative in nature—i.e., interview data, descriptive survey data, and responses to open-ended survey questions. Data were separated into two main themes—‘positive findings’ (i.e., positive aspects and benefits of having a companion animal during the pandemic and perceived positive effects on caregiver and animal well-being) and ‘negative findings’ (i.e., challenges and concerns relating to having a companion animal during the pandemic, and perceived negative effects on caregiver and animal well-being). Within these two themes, we again used codes to organize the data and allow us to spot patterns in the data. For example, data relating to perceived benefits of animal companionship for human well-being during the pandemic were coded ‘Benefits for humans’, and we then identified further sub-themes within this domain such as ‘psychological benefits’, ‘psychosocial benefits’, and ‘health-related benefits’. Data that did not fit into these themes and were not the main focus of the review but which were nevertheless deemed important to the topic were coded as ‘other’ and synthesized separately. 

## 3. Results

**Figure 1:**
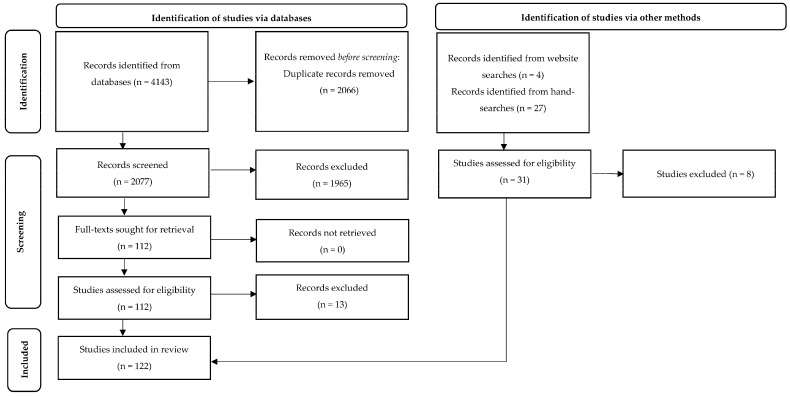
Flow diagram of screening process.

Database searches yielded 4143 citations, of which 2066 were duplicates and immediately excluded. Title screening led to the exclusion of 1864 citations, and abstract screening led to a further 101 being excluded. Full texts of the remaining 112 citations were located, and the articles were read in their entirety to assess whether they met all inclusion criteria. In total, 99 citations found via database searches met the inclusion criteria and were included in the review. Four citations were found from searching for reports published by animal charities, all of which were also included in the review. Hand-searching of reference lists yielded 27 additional citations for screening, of which 19 met the inclusion criteria. Taking the database and hand-searches together, a total of 122 studies were included in the review [73,74,75,76,77,78,79,80,81,82,83,84,85,86,87,88,89,90,91,92,93,94,95,96,97,98,99,100,101,102,103,104,105,106,107,108,109,110,111,112,113,114,115,116,117,118,119,120,121,122,123,124,125,126,127,128,129,130,131,132,133,134,135,136,137,138,139,140,141,142,143,144,145,146,147,148,149,150,151,152,153,154,155,156,157,158,159,160,161,162,163,164,165,166,167,168,169,170,171,172,173,174,175,176,177,178,179,180,181,182,183,184,185,186,187,188,189,190,191,192,193,194], and 21 were excluded based on full-text screening [7,195,196,197,198,199,200,201,202,203,204,205,206,207,208,209,210,211,212,213,214]. A PRISMA flow diagram of the screening process is presented in Figure 1. A list of studies excluded after full-text screening, with reasons, is presented in Appendix B. 

Characteristics of the 122 included studies are presented in Supplementary File S2: Supplementary Table S1. Note that although we have opted to use the terms ‘companion animals’ and ‘caregivers’ throughout the manuscript to avoid using language which suggests that animals are ‘property’ [215], studies included in the review used other terms (e.g., ‘pets’, ‘owners’) and while our terminology is consistent within the manuscript itself, various terms are used throughout the table.

Many different continents and countries were represented in the reviewed studies. Twenty studies had international samples. From North America, we included 22 studies from the United States of America, eight from Canada, and one with participants from both the United States and Canada. Fewer studies were from South America: Brazil (*n* = 2) and Peru (*n* = 1). From Europe, we found studies from the United Kingdom (*n* = 23), Italy (*n* = 4), Spain (*n* = 3), Portugal (*n* = 2), the Netherlands (*n* = 2), Ireland (*n* = 1), Germany (*n* = 1), Lithuania (*n* = 1) and Serbia (*n* = 1). One study included participants from both the United Kingdom and Italy, and one included participants from Belgium, the Netherlands, and the United Kingdom. Additionally, one study included participants from both Spain and Costa Rica. From Oceania, we included nine studies from Australia and three from New Zealand. From Asia, we included studies from China, India, Israel, Japan, Korea, Malaysia, Qatar, and Singapore (*n* = 1 each). Only one study was from Africa (South Africa). Six studies did not specify where participants were from. 

The majority of studies used a cross-sectional survey design, typically also including open-ended questions for qualitative analysis. A minority used semi-structured qualitative interviews (*n* = 11), and three used both surveys and interviews. Five studies compared data collected during lockdown with pre-pandemic data, one study compared lockdown data with retrospectively-collected pre-lockdown data, and four compared cohorts of ‘pandemic puppies’ (acquired during lockdown) with older cohorts. Eleven studies collected data from the same participants at different time-points throughout the pandemic, and one study collected data at different time-points throughout the pandemic but did not clarify whether the same participants were involved at both time-points. One study combined a survey with electronic diaries; one combined interviews, document analysis, and observational research; and one compared two interventions for dogs and their caregivers. Most were conducted in the middle of the pandemic rather than during the recovery period. Many collected their data during the first half of 2020 (*n* = 42), the second half of 2020 (*n* = 15), or throughout both the first and second halves of 2020 (*n* = 16). Ten collected data in the first half of 2021, three collected data in the second half of 2021, and four collected data throughout both halves of 2021. Other periods of data collection included 2022 (*n* = 4), throughout both 2020 and 2021 (*n* = 6), and throughout both 2021 and 2022 (*n* = 1). Two studies collected cross-sectional data at two different time-points in 2020 and 2021. Four studies followed up with participants longitudinally throughout 2020, three followed up with participants from 2020 to 2021, and one followed up with participants from 2021 to 2022. The remaining studies (*n* = 11) did not report the time-points of data collection other than to say that data was collected ‘during the pandemic’ or ‘during lockdown’. 

Many studies (*n* = 37) included caregivers of any type of companion animal. Others focused on dogs (*n* = 32), cats (*n* = 6), both dogs and cats (*n* = 21), horses (*n* = 2) and fish (*n* = 1). Twenty-three studies did not report which types of companion animals were included. Study population sizes ranged from 4–12,068 (mean: 1686; median: 611). Most studies recruited participants from the general population, although some focused on specific sub-groups, including people living alone [133,153]; older adults [85,94,108,110,180,192]; adolescents [137,146,147], both children and adolescents [193], both parents and adolescents [95] or both parents and children [117]; parents [80,81,82,91,143,171]; teleworkers [119,125,172]; employees [183]; university students [111,149] or university students with emotional support animals [126]; individuals identifying as sexual or gender minorities [141]; people with severe mental illness [178]; people with dementia and their caregivers [166]; AIDS survivors [114]; unhoused individuals [105]; people with experience of veterinary consultations during the pandemic [92,93]; people who met the criteria for low-income veterinary care support [145]; people who had suffered the loss of an animal during the pandemic [150]; people who had purchased dogs during lockdown [113,157,158,188]; and domestic abuse helpline staff [118]. Some studies included both general population samples and specific sub-group samples, including people with multiple sclerosis [154], autistic people [155], and people with (dis)abilities [189]. 

### 3.1. Impact of Companion Animals on Human Well-Being 

The first part of our analysis involved collating all quantitative data, which examined being a caregiver of a companion animal as a predictor of any aspect of human well-being. Findings were mixed, with studies showing both positive and negative impacts of animal companionship, as well as many showing no association between animal companionship and well-being. Table 2 illustrates the positive, negative, and neutral findings across the data.

In terms of anxiety, depression, and distress/stress, the findings were extremely mixed. Two studies showed significantly lower anxiety in companion animal caregivers than people without companion animals during the pandemic [109,110], and three studies found significantly higher anxiety in people with companion animals [97,101,134]—although in two of these, this association was found in univariate analysis only [97,101]. A further six studies found no association between animal companionship and anxiety [111,112,116,136,138,173]. Three studies found people with companion animals had significantly lower levels of depression than people without [85,109,138]; in one study, this association was true for people with dogs only [85]. Two studies found people with companion animals had significantly greater depression [97,134], although in one study [97], this association disappeared in multivariate analysis. Seven studies found no association between animal companionship and depression [85,111,112,116,136,173,185]. One study found animal caregivers had significantly lower levels of distress [100], but three studies found caregivers had significantly greater stress [101,147,151]; in one study [101], this association was found in univariate analysis only, and in another [151] this association was for cat caregivers only. A further six studies found no association between animal companionship and stress [111,112,116,151,173,185].

We also found mixed evidence (i.e., evidence suggesting both positive and negative associations) between being a companion animal caregiver and overall mental health, general well-being, positive emotions/positive affect, and loneliness. Regarding overall mental health, one study [165] found people with companion animals reported significantly smaller declines in mental health during lockdown than people without animals, but two studies reported negative findings. Denis-Robichaud et al. [101] found that people with companion animals had significantly poorer mental health, although this association lost significance in multivariate analysis, while one study [178] found people with companion animals experienced significantly greater declines in mental health during the pandemic. Regarding general well-being, studies reported significantly greater well-being in people with companion animals [100,112], significantly poorer well-being in people with companion animals [74], or no relationship between well-being and animal companionship [78,116,132]. Two studies found significantly greater positive emotions/positive affect were reported in people with companion animals [112,125], but one study found that caregivers of non-dog animals had significantly lower positive affect [147] while being caregivers of a dog had no association with positive affect. A further three studies also found no association between animal companionship and positive affect [119,139,185]. As for loneliness, people with companion animals reported significantly lower loneliness in several studies [133,139,165,180], and participants with dogs only reported significantly lower loneliness in another study [153]. However, people with companion animals reported significantly greater loneliness in three studies [74,101,146], although this association was lost in multivariate analysis in one study [101]. There was no association between loneliness and animal companionship in several other studies [78,105,109,162,185] or between loneliness and having a cat [153]. 

There were several well-being outcomes that showed either positive or neutral associations (i.e., no negative associations were found). Evidence relating to the association between animal companionship and post-traumatic growth showed either a significantly positive association (i.e., animal companionship associated with a better outcome) [103] or no association [104]. Healthy coping behaviors were reported to be significantly greater for people with dogs [147], but in the same study, there was no association between coping behaviors and having other animals. Perceived social support was either significantly better for people with companion animals [138] or not related to animal companionship [104]. Regarding the amount of physical activity, one study of people with dogs compared to people without dogs found that those with dogs reported significantly more exercise [119]. One study found that people with dogs were significantly more likely to have a healthy walking routine, but there was no association between having a walking routine and caring for any other type of animal [147]. One study found that having a companion animal was associated with significantly greater low-intensity activity but not moderate or vigorous activity [179], and one study found no association between animal companionship and exercise at all [109]. 

Evidence relating to the association between animal companionship and overall general health, quality of life, and mindfulness suggested either a negative association (i.e., animal companionship associated with poorer outcomes) or no association. Overall general health was significantly poorer for people with companion animals in one study [101], although the association lost significance in multivariate analysis and was not associated with animal companionship in one study [179]. Quality of life was significantly poorer for people with companion animals in two studies [101,162], although the association lost significance in multivariate analysis in one study [101] and was not associated with animal companionship in one study [154]. In one study [153], having a cat was significantly associated with lower mindfulness, but having a dog was not associated with mindfulness. 

Other well-being outcomes did not have mixed (i.e., a combination of positive, negative, and/or neutral) findings, but these outcomes tended to be explored in only a small number of studies, so the findings should be interpreted with caution. We found some evidence that having a companion animal was significantly related to lower tension-anxiety [149] (dog caregivers only), greater emotional well-being [171,179], higher coping self-efficacy [112], lower emotional loneliness caused by deficits in romantic relationships [151], lower isolation [100,114,180], increased socializing [119] (dog caregivers only), greater social functioning [179], greater energy [179], greater perceived positive effects of remote working [125], better self-reported job performance [125] and more time spent outside in the fresh air [143,147] (dog caregivers only, in both studies). 

More negatively, we found evidence that having a companion animal was associated with lower life satisfaction [74], lower presence of life meaning (i.e., a sense of purpose in life) [74], reduced likelihood of spending time with family or exercising as a coping strategy [146] and higher COVID-related impacts [74]. There were also a number of null associations: we found no significant evidence of an association between animal companionship and physical health [178], happiness [138], optimism [78], negative emotions/negative affect [112,116,119,139], self-efficacy [154], resilience [78,112,162], disruption of core beliefs [104], emotional loneliness caused by a deficit of family relationships [151], social loneliness caused by lack of friendships [151], social connectedness [132,180], satisfaction with social roles [154], vitality [74], coronavirus anxiety [132], perceived difficulties of the pandemic [149] or basic psychological need satisfaction [78].

One quantitative study [191] was not included in this analysis due to contradictory results reported. The data presented in the study suggests that animal caregivers had greater levels of insomnia, excessive sleep, lack of enthusiasm, and fears of COVID-19 infection, but the same study also reports that animals reduced insomnia, excessive sleep, anxiety, depression, fatigue, inattention, uncertainty and worry, and increased enthusiasm. Due to the lack of clarity in the results, it was not included in the synthesis presented in Table 2; however, other findings from the study are included in the synthesis of data relating to factors affecting the relationship between animal companionship and well-being.

### 3.2. Factors Affecting the Association between Companion Animals and Caregiver Well-Being 

The second part of our analysis focused again on the quantitative data, this time selecting studies that had used multivariate analysis to investigate factors mediating or moderating the association between animal companionship and human well-being. A number of variables were examined in the included studies, but again, findings were often mixed. 

#### 3.2.1. Socio-Demographic Factors

Gender: Hart et al. [114] found that males with dogs reported feeling alone significantly less than males without dogs and also reported greater human social support, but this effect was not found for females. Amiot et al. [74] found that having a companion animal was significantly associated with lower well-being (including lower vitality, higher loneliness, lower life satisfaction, lower presence of life, and higher COVID-19 impacts) in females but not males. Conversely, Kogan et al. [127] found that female caregivers were significantly more likely to self-report that their companion animal decreased anxiety, depression, feelings of being overwhelmed, feelings of isolation and loneliness, and also more likely than males to report that their companion animals helped them maintain a regular schedule, helped them cope with uncertainty, gave them a sense of purpose or meaning to life and increased self-compassion. 

Age: Amiot et al. [74] found that adult and senior animal caregivers experienced significantly higher COVID-19 impacts than non-caregivers, but the reverse was true for young adults (aged 18–24), suggesting having an animal companion may buffer against the stress of the pandemic for younger people only. Similarly, Kogan et al. [127] found that compared to caregivers aged 39 or younger, caregivers aged 40 and over were significantly less likely to report a positive impact on anxiety, depression, feeling overwhelmed, feeling isolated, loneliness, ability to maintain a regular schedule, ability to deal with uncertainty, and self-compassion. However, Tan et al. [179] found that greater emotional well-being was only seen in animal caregivers who were over the age of 29. 

Nationality: Tan et al. [179] found that non-Chinese animal caregivers had significantly higher emotional well-being, energy, and social functioning during the pandemic than Chinese animal caregivers. 

Marital status: Married people with companion animals reported significantly greater emotional well-being, energy, and social functioning than non-married people with companion animals [179]. 

Employment status: Amiot et al. [74] found that having a companion animal was significantly associated with lower life satisfaction and lower meaning of life for people who were unemployed, students, homemakers, or retired; however, employed people with and without companion animals did not differ in life satisfaction or meaning of life. However, Tan et al. [179] found that employed people with companion animals had significantly higher emotional well-being, energy, and social functioning than those who were not employed. 

Income: In Amiot et al.’s study [74], animal caregivers in the 100–199 k (Canadian dollars) income category reported significantly higher loneliness than non-caregivers, but people with and without companion animals did not differ in loneliness if they earned less than 100 k or more than 200 k. In the same study, animal caregivers in the 0–99 k income bracket experienced significantly higher COVID-19 impacts than non-caregivers in this category, but caregivers and non-caregivers who earned over 100 k did not differ in COVID-19 impacts. Applebaum et al. [75] found that participants with lower annual income were more likely to express concerns relating to how animal companions affected the family unit. 

#### 3.2.2. Factors Relating to Living Situation

Residents of the home: animal caregivers with three or more children living at home reported significantly higher loneliness and greater stress than non-caregivers with three or more children; however, there were no differences in loneliness or stress between caregivers and non-caregivers who had no children, one child or two children [74]. However, in Jeserski et al.’s [122] study, having children in the household had no effect on whether participants described the advantages of having a cat during the pandemic. Martinez-Caja et al. [139] found that those who lived alone showed a significantly greater association between animal attachment and positive affect but also a greater association between animal attachment and negative affect. Xin et al. [191] found that animal caregivers who lived with family had significantly lower depression and anxiety than those who lived with no other humans. Kogan et al. [127] found that caregivers living alone were significantly more likely to report that their companion animals helped with depression than those living with other adults, as well as more likely to report a positive impact of their animal on their sense of purpose or meaning in life. We note that this finding was supported by qualitative findings in other studies: for example, Clements et al. [97] found that individuals living alone particularly appreciated the support from their companion animals, and Bussolari et al.’s [90] participants who lived alone described their dogs as ‘lifesavers’ who improved their mental health by giving them purpose, making them feel less alone, helping them maintain a routine and giving them something to focus on.

Dwelling type: In Amiot et al.’s study [74], animal caregivers living in an apartment or condominium had significantly lower vitality than people without companion animals, but animal companionship had no impact on vitality for those who lived in houses. In the same study, animal caregivers living in the city and countryside reported a lower presence of life than non-caregivers, but there was no such difference for those living in the suburbs. Tan et al. [179] found that animal caregivers who lived in one-to-five-room flats (as opposed to executive flats, condominiums, other apartments, or landed property) reported significantly greater emotional well-being, energy, and social functioning. However, the authors do not make clear the ways in which the flats differ from executive flats, condominiums, apartments, or landed property. 

Rurality: Lima et al. [136] found that for people living in rural and semi-urban areas, having a dog was significantly associated with lower anxiety, while people with dogs living in urban areas were significantly more anxious than people without dogs. 

Number of animals in the house: The number of companion animals was not associated with well-being in one study [74]. However, Xin et al.’s data [191] suggested that people with more than one companion animal reported significantly lower depression than those with only one animal.

#### 3.2.3. Factors Relating to Humans

Resilience: Barklam and Felisberti [78] found that among people with low resilience, having a companion animal was linked to significantly higher levels of positive feelings and affect balance (the latter being calculated by subtracting the ‘negative feelings’ score from the ‘positive feelings’ score), but among people with high resilience, having a companion animal was linked to greater negative feelings and lower affect balance. There was no interaction between having a companion animal and resilience in the prediction of overall well-being.

Mental health: Falck et al. [107] found that among people without anxiety disorders, people with companion animals had significantly greater depression and anxiety than people without animals. Among people without depression or mood disorders, there were no differences in depression scores between people with and without animals. Among people with any mental health disorder, people with animals had significantly greater depression than people without. Overall, the findings of this study suggested that having a companion animal was not associated with well-being in people with no mental health disorders but that companion animals might worsen anxiety and depression symptoms for those with mental health disorders. The authors suggested that the challenges associated with caring for animals during the pandemic might not substantially affect the well-being of people without mental health conditions, but the additional burden of animal care may contribute to poorer outcomes for those with mental disorders. 

Neurodiverse conditions: Oomen et al. [155] found that autistic people were significantly more likely to feel worried about their animals during the pandemic than non-autistic people. 

Long-COVID: Among participants with long-COVID only, there was a significant negative association between animal-related concerns (and responsibility-related concerns) and overall quality of life [131]. In the same study, the human–animal relationship for the long-COVID group was significantly associated with poorer quality of life, poorer psychological health, and greater symptoms of depression. The participants who had not been infected with COVID-19 showed no association between human–animal relationships and any aspect of well-being. 

Socialization: Companion animal caregivers who met up with others more frequently had significantly greater positive emotions and less loneliness than those who did not [185]. 

Concerns for animal rights/animal welfare: Among animal caregivers with high resilience only, stronger concerns for animal rights and animal welfare were linked to significantly greater negative feelings and lower well-being [78]. 

#### 3.2.4. Factors Relating to Animals

Type of companion animal: Several studies found that people with dogs had better well-being than those with other animals: compared to people with other animals, dog caregivers felt significantly better supported [114] and had significantly higher vitality [74], greater life satisfaction [74] and greater quality of life [87], as well as significantly lower levels of loneliness [74,114,151], larger reductions in loneliness between lockdown and recovery [151], larger reductions in stress between lockdown and recovery [151], less isolation [114,127], lower levels of insomnia [191], lower fears of COVID-19 infection [191], less sadness [114], less stress [151] and lower COVID-related impacts [74]. Dog caregivers were also significantly more likely to report that their animal helped them maintain a regular schedule [127]. Ogata et al. [151] found that cat caregivers had significantly higher stress than both dog caregivers and people without companion animals. Conversely, Grajfoner et al. [112] found that cat caregivers had significantly higher psychological well-being and greater positive emotions than dog caregivers. Hoffman [119] found that dog caregivers were significantly more likely than cat caregivers to report that work life and personal life interfered with each other and that family members created distractions while remote working. Martinez-Caja et al. [139] found that people with horses had significantly greater positive affect than people with dogs, cats, rabbits, and birds. Others found no significant differences: Xin et al. [191] found no difference between cat and dog caregivers in terms of excessive sleep, anxiety, depression, fatigue, enthusiasm, or attention. Kogan et al. [127] found that animal species did not predict depression, loneliness, feeling overwhelmed, ability to deal with uncertainty or self-compassion. Martinez-Caja et al. [139] found no association between companion animal species and negative affect, and Mueller et al. [146] found no association between animal species and loneliness. 

Length of time being an animal caregiver: Length of time as a companion animal caregiver was not associated with well-being [78]. 

Time spent with animals: In Barklam and Felisberti’s study [78], animal caregivers who reported an increase in time spent actively playing with their animals since the start of the pandemic had significantly higher well-being and more positive feelings than those who reported no increase. Tan et al. [179] found that those who were the main caregivers for their animals reported significantly greater emotional well-being, energy, and social functioning during the pandemic than those who were not. However, Clements et al. [97] reported that greater engagement with dogs was associated with significantly poorer mental well-being. In the same study, greater engagement with cats was associated with both significantly lower anxiety and greater depression, while engagement with ornamental fish was not associated with any well-being outcomes. Shoesmith et al. [178] found that engagement with animals was not associated with changes in either physical or mental health during the pandemic. 

Walking dogs: Walking dogs at least once a day off-set increases loneliness among older adults who experienced high social consequences related to the pandemic [94]. Dog caregivers who increased the frequency and/or duration of walks with their dogs since the start of the pandemic were found to have significantly higher well-being, more positive feelings, and higher affect balance than people with other animals [78]. Lima et al. [136] found that walking dogs was marginally associated with lower anxiety. Clements et al. [97] found that walking dogs for less time than average per day was associated with significantly greater anxiety and loneliness. In Lee et al.’s study [135], a greater frequency of dog walking was significantly associated with better health for both caregivers and dogs, and there was an indirect effect of dog walking on loneliness, partially mediated by attachment to the dog. However, Lau and Oliva [133] found that dog-walking was not associated with loneliness or mindfulness, and Zaninotto et al. [194] found that dog-walking was associated with fewer negative psychological symptoms in caregivers during the last (fourth) week of their lockdown study only. 

Perceived costs of pet ownership: Lima et al. [136] found that dog caregivers who reported higher perceived costs (i.e., challenges) of having a dog reported significantly higher anxiety and depression.

Animals’ effect on emotional experience: Bennett et al. [79] reported that participants who perceived companion animals to have a negative effect on their emotions had significantly poorer life satisfaction, while participants who perceived animals to have a positive effect on their emotions had better general well-being.

Changes in animal welfare/behavior: Shoesmith et al. [176] found that poorer mental health was significantly associated with more reported positive changes in companion animal welfare and behavior. 

#### 3.2.5. Factors Relating to Human-Animal Relationships

Support from animals: Bowen et al. [87] found that for every one-point improvement in animal caregiver quality of life during lockdown, the participant was 34.5% more likely to report low support from their companion animal. The same study reported that the more a companion animal was perceived as providing comfort, the more likely the caregiver was to report poorer quality of life. However, Bennett et al. [79] reported that higher emotional support from animals predicted significantly poorer life satisfaction and poorer well-being.

Worries about animals: Bennetts et al. [82] found that participants who were worried about their animals (in terms of their care, well-being, or behavior) during the pandemic were more likely to report psychological distress.

Attachment to companion animal: Many studies found that the level of attachment to the companion animal affected caregivers’ well-being—but again, we found a combination of positive, negative, and neutral associations. Positively, greater attachment to animals was significantly associated with reductions in anxiety [127], reductions in depression [127], greater physical activity [179], better emotional well-being [179], better mood [149], greater prosocial behavior [117], better emotion regulation [117], higher post-traumatic growth [104], greater positive affect [139], better general health [179], lower likelihood of feeling overwhelmed [127], lower isolation [127], lower loneliness [127], fewer conduct problems [117], less hyperactivity [117], less confusion-bewilderment [149], less fatigue-inertia [149], less negativity [117], a greater sense of purpose or meaning in life [127], more vigor-activity [149] and higher energy levels [179]. Ratschen et al. [165] found that higher companion animal attachment was significantly associated with better mental health pre-lockdown but not during lockdown (although scores were approaching significance). Attachment to companion animals moderated the effects on telework of self-reported job performance via positive affect, with the relationship becoming stronger for those who were more attached to their animals [125]. Barklam and Felisberti [78] found that among caregivers with low resilience, greater attachment was associated with more positive feelings; however, attachment was not associated with optimism or satisfaction with basic psychological needs in the prediction of well-being. Wan et al. [183] found that attachment buffered the relationships between stress and substance use/psychological strain, with attachment moderating the relationship between stress and alcohol use, marijuana use, emotional exhaustion, and depression (but not cigarette use). In the same study, stress was positively related to alcohol use, marijuana use, emotional exhaustion, and depression only when animal attachment was low. Similarly, the indirect effects of job insecurity on alcohol use, marijuana use, exhaustion, and depression were only significant when attachment to animals was low.

Negatively, higher attachment to companion animals was associated with significantly greater levels of depression [136,185], greater anxiety [80,136], greater worry [80], lower positive moods/affect [139,185], greater loneliness [185], greater distress [80], greater disruption of core beliefs (e.g., re-examining beliefs about the fairness of life, due to the COVID-19 pandemic) [103,104] and greater COVID-specific worries [80]. We note that Bennetts et al. [80] suggested that the negative associations between animal attachment and well-being in their study attenuated somewhat after controlling for mental health. The authors pointed out that it was not clear whether people who were feeling unsettled were gravitating towards their animals for comfort or whether stronger pet attachment contributed to their distress and suggested the reality may be a combination of the two. Participants may have turned to their animals as a source of comfort due to traditional social supports being less accessible; strong attachment to animals is also likely to reflect high empathy, which might increase vulnerability to distress. They suggested longitudinal research is needed to better understand the mechanisms underpinning animal attachment and caregiver mental health. 

Dominick et al. [104] suggested that the lack of differences in well-being between people with and without animals, as well as the association between animal attachment and post-traumatic growth and core belief disruption, implies that attachment plays a greater role in well-being than simply having a companion animal or not.

However, there were also neutral findings, suggesting no association between attachment to animals and social functioning [179], stress [185], depression [138], anxiety [138], ability to cope with uncertainty [127], happiness [138], peer problems [117], changes in loneliness over the pandemic [146] or overall well-being [78,132]. 

Additionally, one study [142] found that the relationship between attachment to animals and mental health differed depending on the severity of mental health symptoms. Attachment was a protective factor for individuals with moderate and high levels of mental health symptoms and predicted transition to a less severe symptom profile, but individuals with high attachment and severe symptom profiles fared worse. Hawkins and Brodie [116] reported mixed findings, with people highly attached to their animals reporting significantly lower psychological well-being and higher depression, anxiety, stress, and negative affect at the first point of data collection. However, two weeks later, only negative affect was significantly associated with animal attachment and highly attached caregivers showed a reduction in anxiety and additionally a reduction in negative affect after another two weeks. Less attached animal caregivers showed an increase in negative affect and anxiety, and the authors concluded that animal attachment may have caused hardships earlier in the pandemic, which negatively affected mental health, but as restrictions began to lift, animal attachment was beneficial for reducing negative emotions. 

### 3.3. Perceived Benefits of Companion Animals for Their Caregivers during the Pandemic and Benefits of the Pandemic for Animals

Despite the inconsistent findings relating to the association between companion animals and caregiver well-being, several studies found that when participants were asked whether they believed that their animals positively affected their well-being during the pandemic, the majority reported that animals were beneficial to their mental health and improved the lockdown experience [73,77,79,82,88,90,124,153,181]. Participants reported a wide variety of benefits to having companion animals during the COVID-19 pandemic; additionally, a number of positive effects of the pandemic on animals themselves were noted. Qualitative data from interview studies and open-ended survey questions revealed a number of themes relating to positive experiences during the pandemic. An overview of the themes relating to positive findings is presented in Table 3.

#### 3.3.1. Psychological Benefits for Humans

The first theme we identified related to the psychological benefits of animal companionship during the pandemic for their human caregivers. Participants in seventeen studies described their animals as reducing stress, tension, or distress [73,81,86,90,98,108,122,123,126,130,131,146,149,177,181,188,192], while twelve studies reported that caregivers perceived their animals to improve their mental health and well-being [78,90,97,98,102,121,127,154,156,177,178,181]. There were a number of ways in which animal companionship was perceived to have improved well-being. For example, in twenty studies, animals were reported to provide routine and a sense of structure, helping caregivers to feel ‘normal’ in a very abnormal and uncertain time [73,77,79,81,86,90,97,98,102,121,126,127,153,156,166,177,178,181,184,192]. Fourteen studies described animals as giving participants’ lives purpose and meaning [79,81,90,97,98,102,121,126,127,153,156,172,178,192]; participants reported that their animals motivated them to get up and do things and prevented them from feeling useless. Participants in thirteen studies described how animals offered a welcome distraction from pandemic-related news and the reality of living through a global pandemic [73,77,81,90,97,98,121,126,156,166,177,181,192]; animals directed attention away from COVID-related stressors and provided something positive to focus on. Nine studies suggested animals helped caregivers to cope with the emotions and uncertainty of the pandemic [79,81,97,98,124,127,146,154,192], while two studies showed participants thought their animals diminished feelings of being overwhelmed [127,172]. Participants in eight studies described the positive impact of animals on the home, suggesting they brought feelings of joy, pleasantness, and coziness to the home environment [73,79,108,166,167,172,181,188]—undoubtedly important during a time when most people were spending more time at home than ever. A further seven studies described the fun, entertainment, and laughter animals brought to the home [81,98,102,137,153,156,178]. Animals were also described as helping caregivers to relax [102,131,167], helping young people feel good [91,193], providing a calming presence [90,98,126,131,137,159,172,177,178,181], reducing the sadness of being away from family [108], providing a sense of perspective [97,156], helping caregivers to feel more grounded [98], providing a reminder to live in the moment [156], improving mood [77,79,102,121,153], improving self-compassion [127], fostering a sense of gratitude [90,98] and boosting morale [95]. 

#### 3.3.2. Psychosocial Benefits for Humans

Companion animals were reported to provide a sense of companionship, which was a valuable alternative to human interpersonal connections [77,81,90,95,97,102,153,156,177,178,181,184,192]. This was found to be particularly important for parents of children with no siblings [81]. Participants in eight studies described animals as providing psychological or emotional support [77,79,97,166,172,177,181,184]. This was particularly important for those who had experienced negative impacts of the pandemic (such as being furloughed) [184]. Participants in eighteen studies described animals as providing comfort and love [73,78,79,81,90,97,98,121,126,131,140,156,172,177,178,181,188,192]. In eight studies, participants suggested that physical touch with animals provided a comforting substitute for physical contact with humans [73,77,90,97,153,156,177,181]. Fourteen studies found that animals were perceived to reduce feelings of isolation and loneliness [73,90,98,121,126,127,153,156,166,172,177,178,188,192], and three studies reported animals provided a sense of safety, security, and protection during the pandemic [166,172,181]. In ten studies, animals were described as ‘social catalysts’ inviting interactions with other people [79,81,97,102,121,153,156,166,172,192], for example, encouraging dog caregivers to talk to one another on walks or being ‘conversation starters’ on video calls during remote work/remote studying. These animal-inspired interactions were sometimes the only human interaction people would receive during lockdown [192]. One study also suggested that caregivers felt encouraged to reach out and support other animal caregivers—for example, by sharing advice online regarding how to support animal well-being during the pandemic [190]. Finally, two studies found that participants reported deriving pleasure from having animals to look after/care for during the pandemic [108,192]. 

#### 3.3.3. Health-Related Benefits for Humans

Seventeen studies described how animals encouraged their caregivers to engage with nature and the outdoors [77,81,86,90,102,106,121,131,143,153,154,156,166,177,178,184,192]. Caregivers in these studies were mostly dog caregivers, and they described how exercising their animals helped them to increase their own exercise, stay fit, and spend time outdoors in the fresh air. 

#### 3.3.4. Work-Related Benefits for Humans 

A small number of studies reported on how animals positively affected remote working. Participants in four studies described how their animals encouraged them to take breaks from their computers while working from home [102,156,172,181], and one study suggested that animals encouraged caregivers to have a better work–life balance [86]. One study also suggested that animals were perceived to reduce work stress, improve productivity, and increase motivation to work [172]. 

#### 3.3.5. Other Benefits for Humans

Participants in one study [190] described how they were encouraged to look into financial assistance programs as a result of being concerned about how financial loss would affect their ability to seek veterinary care for their animals. As a result, they learned about assistance programs available and were able to take advantage of these. 

#### 3.3.6. Benefits for Human-Animal Relationships

Thirteen studies reported increased emotional bonds between caregivers and their companion animals [87,88,90,98,101,102,127,128,129,135,140,153,168], while participants in seventeen studies described increased companionship and quality time spent interacting with their animals [73,78,86,87,88,90,96,98,102,128,129,135,153,167,168,176,190]. Five studies reported on the ability of humans and animals to read each other’s body language [90,177,181,190,192]; this ability was perceived to have improved during the pandemic, and animals were seen as attuned to their caregivers’ moods and emotional needs and actively sought to help them feel better. Two studies found the perceived costs (challenges) of companion animals were reduced during the pandemic [88,99], and two studies found appreciation for companion animals increased [177,178]. Two studies suggested lockdown provided time for children to develop their caring skills and take more responsibility for animals [73,193], and one study suggested that lockdown provided an opportunity for children to better understand animals’ boundaries [73]. 

#### 3.3.7. Benefits of the Pandemic for Animals’ Well-Being

Animals were perceived to be happier, perhaps due to increased time with families at home [90,106,126,153,159], and were perceived to be less anxious about their caregivers leaving them alone [102]. Three studies reported that animals were seen to enjoy increased companionship [84,106,121], and one study suggested they received more stimulation [106]. Animals were also perceived to be calmer/more relaxed in ten studies [84,87,102,106,122,123,144,153,168,176], more playful [122,123,176], more affectionate [90,106,140,176], less reactive [84] and less stressed [164] (with the exception of cats, who were not less stressed). One study suggested behavioral problems were reduced [164]. Animals’ physical health was not typically perceived to have changed, although Woolley et al. [187] did report decreased odds of episodes of coughing in dogs. Six studies suggested animals got more exercise [84,102,106,135,153,187], and three studies suggested there were more opportunities for training [102,106,176]. Walks for dogs were described to be more pleasant for reactive dogs, as there were fewer people around [102,156] and safer due to less traffic [106]. Finally, aquarium keepers described how their aquariums were better maintained during the pandemic and had more money spent on them [130]. 

#### 3.3.8. Advantages of Veterinary Telemedicine

A small number of studies identified positive aspects of remote veterinary appointments. These were sometimes perceived as less stressful for both animals and caregivers [93], quicker [93], cheaper [93] and more convenient [93]. Participants in the same study perceived telemedicine to be advantageous in terms of removing the need for transportation and time spent in waiting rooms, as well as safer. Participants in two studies expressed appreciation of how their veterinarians communicated with them about COVID-19 transmission and extra safety precautions taken [128,129]. 

### 3.4. Perceived Challenges of Companion Animals for Their Caregivers during the Pandemic and Challenges of the Pandemic for Animals

A number of challenges and negative aspects of animal companionship during the pandemic were also noted, as well as the negative impacts of the pandemic on animals themselves. An overview of the themes relating to negative findings is presented in Table 4.

#### 3.4.1. Concerns about Meeting Animals’ Basic Needs

Many of the challenges reported by participants related to meeting the basic needs of their companion animals during the pandemic. For example, the restrictions put in place due to the pandemic caused worries about procuring food [75,77,79,90,98,102,154,156,165,169]; this concern was particularly salient for those with animals with specific medical or dietary needs [79]. Participants also worried about procuring other animal care supplies such as cat litter, toys, leashes, beds, and bowls [75,77,79,81,97,106,154,156,176], and participants in five studies reported concerns about other people panic buying and hoarding supplies, leaving none left for others [75,77,79,98,156]. Participants in three studies specifically described fears of not being able to access medication for their animals [75,87,156] and twenty-seven studies described difficulties accessing, or concerns about accessing, veterinary care [75,77,79,81,86,87,90,97,98,102,106,121,122,123,126,128,129,145,154,156,165,177,178,184,186,188,190]. Due to concerns about access to veterinary care, some reported concerns about vaccinations lapsing [184]; indeed, Woolley et al. [187] found reduced rates of vaccination during lockdown. Three studies described concerns about reduced access to professional grooming services [75,97,156], and participants in ten studies reported challenges around walking/exercising animals [81,87,97,106,121,131,140,154,165,176]. 

#### 3.4.2. Concerns about Meeting Animals’ Social and Behavioral Needs

Participants described concerns about animals not getting enough enrichment/stimulation [75,90,102,121] or physical touch [90]. Participants also reported concerns about their animals missing out on day-care [75,90,156], training [75,81,102,140,156] and socialization [75,77,81,90,102,121,140,156]. Dog caregivers described concerns about the loss of service dogs or therapy dog activities [90,176], dog sports and play activities [99], and access to professional dog-walkers [156]. Participants in one study reported worries about a lack of control over their animals’ routines [184]. Participants reported concerns about animals developing behavioral issues [90,121] or worsening chronic behavioral problems [75] and the need to retrain animals in the future [121]. In one study, participants described difficulties balancing adherence to public health guidelines with meeting their animals’ needs [156]. 

#### 3.4.3. COVID-Related Concerns 

Participants in sixteen studies expressed concerns about what would happen to their animals if caregivers themselves were ill, incapacitated, or hospitalized due to COVID-19 [75,76,77,79,81,86,90,98,102,121,128,129,131,154,165,186]. Participants in three studies reported that they would delay or avoid testing or treatment for COVID-19 due to concern about their animals [76,102,141]. Applebaum et al. [76] found that approximately one-tenth (*n* = 122) of their sample were either uncertain about or would indeed delay or avoid testing for COVID-19 due to concerns for companion animals’ welfare. Over one-tenth (*n* = 168) were either uncertain or would definitely delay or avoid treatment for COVID-19 for the same reason. In the same study, willingness to delay or avoid testing or treatment was predicted by attachment to companion animals; that is, people who were highly attached to their animals were more likely to risk their own health to avoid separation from their animals. Most suggested they would not seek healthcare before securing accommodations for their animals, and many were still wary about seeking healthcare due to concerns about the quality of care in their absence, especially those with animals who had special care needs. More than a third did not have a plan in place for if they became ill. Dogs Trust [102] found that 65% of people indicated they would delay hospital treatment to care for their dogs if needed. Matijczak et al. [141] found that attachment to animals predicted delaying or avoiding COVID-19 treatment. In this study, sexual and gender minority (SGM) participants were more likely to delay or avoid testing when they reported high attachment to their animal and low levels of social support. When participants reported high attachment to animals and high levels of social support, SGM status did not predict intent to delay or avoid testing, nor was there an association between SGM status and delaying or avoiding testing when attachment to animals was low or moderate.

Eleven studies reported concerns about animals catching COVID-19 [75,79,81,86,90,97,98,102,106,177,186], and eight studies reported concerns about humans catching COVID-19 from their animals [75,86,97,102,106,154,177,186]. In one study, the fear of animals being able to catch COVID-19 had also led to fears that it would be recommended that animals should be euthanized if they caught COVID-19, and participants feared losing their animals [98]. Participants in seven studies described concerns about potentially catching COVID-19 while exercising animals, seeking veterinary care, or shopping for animal supplies [75,86,102,121,145,156,189]. For example, participants who walked their dogs described fears of catching COVID-19 from dog waste bins, gates, other dogs, or other people during their walks [156]. Participants in one study were concerned about needing to physically distance themselves from their animals if they caught COVID-19 [75], and participants with long-COVID described how exhausting it was to look after animals when ill [131]. 

#### 3.4.4. Challenges of Remote Working/Studying with Animals in the Home

Participants in six studies described their animals demanding attention while they worked or studied from home [75,77,79,90,172,181], and participants in five studies specified that animals would interrupt their video conferences (e.g., with vocalizations and/or appearing on video calls) [75,81,86,172,181]. In five studies, animals were described as being distracting during work hours [75,119,126,172,181], and in one study, parents were concerned about animals potentially distracting children from their home studies [81]. 

#### 3.4.5. Psychological Challenges for Humans

Participants described various negative psychological states relating to animal companionship during the pandemic, including irritation, frustration, or annoyance at animals [75,131], guilt around being home but not being able to devote their full attention to their animals [90], and reduced mental health caused by missing animals who did not live in the home (e.g., horses) [186]. Participants in five studies reported struggling to balance the competing demands of animal care and other responsibilities, such as caring for family members, home-schooling, or remote working [75,81,90,126,172]. Participants in two studies described fears of their animals picking up on their own stress and their own anxiety potentially exacerbating that of their animals [90,140]. Sixteen studies described worries about how animals would cope when things went back to ‘normal’, lockdown restrictions eased, and caregivers returned to work; in particular, participants feared animals missing the company and attention they had become accustomed to and potentially developing separation anxiety [75,81,84,86,90,102,106,120,121,153,154,156,165,177,178,192]. Fifteen studies described financial concerns, causing participants to worry about how they would care for their animals if they lost their income or were furloughed [75,77,79,81,106,120,126,129,145,154,161,177,186,189]. Participants in eight studies described the emotional challenge of having to wait outside when their animals received veterinary care [79,102,113,128,129,145,189,190]. In some cases, participants actually avoided taking animals for treatment where normally they would have, as they could not bear the thought of their animals having treatment without their caregivers there to accompany them [113]. Participants in one study felt negatively affected by the loss of interactions with other animal caregivers during the pandemic [184]. One study described how being at home with animals in lockdown could cause jealousy in children when animals preferred the company of their parents [73]. Many participants also expressed concerns about companion animals in general (not their own); for example, they expressed concerns about how increased domestic abuse during lockdown might affect animals [106] and were worried about other people’s animals experiencing separation anxiety, reduced exercise, boredom or being abandoned after lockdown [81,102,106,177]. Finally, one study noted that COVID-19 restrictions limited the ability to knock on doors or conduct thorough searches if animals went missing, which could be very stressful for their caregivers [81]. 

#### 3.4.6. Health-Related Challenges for Humans

Participants in one study described how the extra time spent at home with animals exacerbated allergies to animal dander [81]. 

#### 3.4.7. Negative Impacts of the Pandemic on Animal Behavior

The change in behavior reported in the greatest number of studies was increased neediness, which resulted in attention-seeking, insecurity, and clinginess to caregivers [75,77,79,81,84,87,98,102,106,115,121,140,144,153,156,167,168,172,175,176,177]. Eight studies reported increased vocalization from animals [87,99,102,106,156,164,167,175], which was attributed in one study to neighbors making more noise at home and more deliveries coming to the home [156]. Nine studies reported increased nervousness, shyness, or fear [84,87,102,106,126,156,167,170,176]. Separation anxiety was reported in thirteen studies [79,81,83,84,106,115,121,126,160,161,172,175,188]. The PDSA [161] found an increase in behaviors relating to lack of socialization, including fear, aggressiveness, and nervousness. One study [82] reported that a minority of participants felt their animals were unsettled and anxious. Other behavioral changes included increased excitability [84,87], increased frustration [87,102], increased agitation [86], increased restlessness [121,156], increased reactivity [84,156], increased hyperactivity [167], increased stress [81,87,106] and increased stress in cats only [164], increased anxiety [84,176], increased irritability [87,176], increased aggression [84,164,170,175], increased destructive behavior [121,160,167], increased mouthing/nipping [121], increased territoriality [126] and increased toileting accidents inside the home [106,167]. Animals were also described as less social [84,126], more expectant of increased attention [75], and having regressed in training [84]. Participants in two studies described more stress-related behaviors during veterinary appointments [92,148], and one study described general behavioral issues caused by a lack of socialization and training [113]. We note that although a number of negative animal behavioral changes were reported, these tended to be reported by a minority of participants in their respective studies. Indeed, several studies reported that, while behavioral changes in animals were described, the majority of participants felt behavior had not changed—or if behavior had changed, it was mostly in positive ways [99,122,123,176].

#### 3.4.8. Negative Impacts on Animal Health and Well-Being

Participants reported changes in animals’ appetite [86,122], reduced exercise [96,156,164,176,182], weight gain and over-feeding [81,106,160,161,167,176], increased health issues in a minority of animals [122,123] and interrupted sleep or relaxation for animals [86,106]. Managing animals’ weight was particularly a concern with children in the home who enjoyed sharing their food or giving treats [81]. One study described how increased noise in the home due to all family members being at home might be stressful for animals, while another reported that children initiate unwanted interactions with animals [106]. Two studies reported that animals spent less time with caregivers: in one study, this was due to caregivers working increased hours [177], while in another, this was because horses were not kept at home and their caregivers had difficulties accessing them [186]. Dogs were reported to have fewer interactions with other dogs [96,102,106,153,156,167,176,188], and their walks suffered in a number of ways, including being restricted to leashes [106] and less variety in where they could walk [156]. Vučinić et al. [182] reported reduced dog-walking, particularly among older caregivers; a minority walked their dogs more due to having more free time but for the majority, walking time was reduced. In the same study, dog walks were particularly reduced for bigger, older, and higher-energy dogs; in households with two dogs rather than one; and where caregivers rated their relationship with their dog as medium or strong. Walk duration was especially reduced when dog caregivers were vulnerable or living with vulnerable household members and when they lived with others rather than alone [156]. In the same study, participants reported that reasons for continuing to walk included not having anyone else to help; not trusting anyone else with their dogs; living in rural areas or having access to private land; having symptoms in February when little was known about the virus; and believing it was important for mental health as long as extra precautions were taken such as walking early in the morning or late at night to avoid other people. Animals were perceived to have poor car sense due to not being around cars, as there was less traffic on the road during the pandemic [106]. Finally, five studies reported that animals’ routines were disrupted [79,81,106,159,186]. It is important to note that even in studies where many challenges were reported, animals were often perceived to have the same, or better, well-being during the pandemic than pre-pandemic [106,159,168]. 

#### 3.4.9. Challenges of Veterinary Telemedicine

Numerous challenges relating to virtual veterinary appointments were described. The lack of clinical examination was concerning to participants in one study [93], while delays in receiving diagnosis or treatment were also cited as stressful [93], and the risk of misdiagnosis due to veterinarians not being able to see the animals was believed to be a problem by both caregivers and veterinarians [93,113]. Participants in five studies found it more difficult to communicate with veterinarians remotely [93,128,129,145,189], and the experience was perceived to be stressful in one study [93]. Price quotations were not always clear, resulting in discrepancies between the true cost of veterinary appointments and what participants believed they would be paying [189]. Finally, veterinarians were overwhelmed due to the pandemic ‘adoption blitzes’, which led to a surge in demand for care and treatment [148]. 

#### 3.4.10. Animal Loss

A small number of studies discussed the challenges of animal loss during COVID-19 restrictions. Participants in five studies described the heartbreak of not being allowed to be present for euthanasia or concerns that they would not be allowed to be present should their animal require it [75,81,83,128,156]. Participants in one study suggested that fears of animal loss were heightened due to the events of 2020 and having more time to spend dwelling on the issue [81]. Two studies described either the difficulties of having to grieve alone due to social restrictions or fears of having to grieve alone [75,177], while one study suggested that having more time at home made the grieving process more difficult, amplifying the sense of loss and negatively affecting caregivers’ mental health [81]. 

### 3.5. Factors Predicting Changes in Animal Behavior and Well-Being during the Pandemic

Jezierski et al. [123] compared participants in lockdown or quarantine with a ‘control’ group who did not undergo these restrictions and found those in lockdown were 1.8 times more likely to report behavioral changes in their animals, suggesting that lockdown measures may indeed influence animal behaviors. We carried out a thematic analysis of data relating to predictors of animal behavior change during the pandemic in order to identify factors that may affect whether behavior changed or not. 

#### 3.5.1. Animal-Related Factors

*Number of animals in the household*: Cats coped better if there were more cats in the household [87].

*Type of animal*: In some studies, dogs’ overall quality of life was generally perceived to have worsened, while cats were perceived to have improved [87]. Shoesmith et al. [176] found that cats had significantly higher positive changes during the pandemic than dogs, non-mammals, and horses. In the same study, dogs had significantly greater negative changes than cats or small mammals.

*Age of animal*: Harvey et al. [115] found separation-related behaviors to be more common in older dogs. 

*Animals’ appetite*: Platto et al. [164] found behavioral problems to be less common in dogs with good appetites. 

*Pre-existing behavioral problems*: Sherwell et al. [175] found that dogs with any pre-existing signs of separation problems (especially vocalization, self-injury, and chewing to escape confinement) had a greater increase in the number of behavioral issues experienced during lockdown. 

#### 3.5.2. Caregiver-Related Factors

*Caregivers at home*: Dogs in homes with all family members at home were more likely to experience increased behavioral problems [87]. Sherwell et al. [175] found that the change to working from home was initially related to a decreased risk of aggression, but over time, those who continued working from home were at increased risk of aggression from their dogs.

*Number of people at home*: For every additional person aged 18–64 in the household, dogs were 1.4 times more likely to experience worsening problematic vocalizations [87]. Riggio et al. [168] found that caregivers who reported houses to be too small for all household members were more likely to report increased aggression in cats towards other cats. 

*COVID-19 prevention measures*: Jezierski et al. [123] found that those who took more measures to prevent the spread of COVID-19 were also more likely to report changes in their dogs’ behavior.

*Caregiver quality of life/well-being*: Morgan et al. [144] found that behavioral problems in dogs increased when caregivers’ quality of life was more impaired; in the same study, poor quality of life in animals was also associated with impaired quality of life in caregivers. Piotti et al. [163] found that caregivers who perceived their well-being to be worse during lockdown reported poorer scores for their animals’ physical quality of life. In the same study, the only predictor of animals’ psychological quality of life was caregivers’ financial loss, with animals perceived to have a better psychological quality of life when caregivers reported small or no financial losses compared to large losses. 

#### 3.5.3. Human-Animal Relationship Factors

*Human-animal relationships*: Excessive vocalization in dogs during the pandemic was predicted by greater emotional attachment with humans and experiencing greater anger from caregivers [87]. Emotional closeness to caregivers also predicted poor coping in cats [87]. However, Shoesmith et al. [176] found that positive changes in animal welfare and behavior were predicted by stronger human–animal bonds and also by not perceiving companion animals as family members.

#### 3.5.4. Factors Relating to COVID-19-Related Changes

*Change in time spent alone*: Harvey et al. [115] found separation-related behaviors to be more common in dogs who experienced a greater change in time left alone post-lockdown. 

*Exercise*: Bowen et al. [87] found that dogs who had fewer walks per day during the pandemic were more likely to show increased vocalizations, while Platto et al. [164] found behavioral problems in general were more common in dogs who were walked less. 

### 3.6. Other Findings

We also noted various other findings that were not examined in enough studies to warrant separate themes within this review but which are nonetheless important.

#### 3.6.1. Animals Purchased or Adopted during Lockdown 

Seven studies considered new caregivers’ motivations for purchasing or adopting new animals during the pandemic [81,113,157,158,160,161,188]. All studies focused on dogs other than two [160,161], which included dogs, cats, and rabbits. 

While many participants reported that their decisions were unrelated to the timing of the pandemic [81], others were influenced by the pandemic—in fact, more than two in five participants were influenced by the pandemic in Packer et al.’s [157] study. Gregory [113] found evidence that some people impulsively purchased dogs during lockdown despite having performed no research on the breed or their specific needs; in some cases, purchases were made even after being advised not to go ahead with the purchase by animal welfare organizations, which could threaten the well-being of the dogs. Pandemic-related reasons for acquiring a new dog during lockdown included wanting to support the mental or physical health of children [81], challenges adapting to more time at home [188], having extra time at home to help animals settle in, bond with them and train them [81,157,161,188]; wanting to keep children busy [157]; wanting a distraction from the pandemic [157,188]; boredom due to lockdown [157]; wanting a companion and protector while home alone [188]; wanting a reason to go outside and exercise [157]; having extra money due to lockdown [157]; wanting more company [157]; and wanting to benefit participants’ own mental health [81,157]. Non-pandemic-related reasons included wanting children to learn responsibilities [81]; wanting companionship for existing animals [81,188]; or as part of the healing process after the recent loss of an animal [81,188]. 

Comparing people who purchased puppies during lockdown to people who had purchased puppies in 2019, Packer et al. [158] found that lockdown puppy purchasers were more likely to cite exercise encouragement, improving the mental health of themselves and their families, and companionship for children as reasons for purchasing puppies than those who did so in 2019. The 2020 PDSA survey [160] also found that people who purchased animals during lockdown were more likely to report having got their animal for companionship reasons than people who acquired animals pre-pandemic. However, by 2021, people purchasing dogs were less likely to buy dogs to encourage exercise, improve their family’s mental health, or for companionship than those who purchased them in 2020, although these motivations were still higher than in 2019 [158]. 

Several studies found that newly acquired animals (specifically dogs) experienced behavioral problems. Gregory [113] found mature dogs were better able to adapt to the changes brought about by the pandemic than adolescent dogs and puppies, as they had already been exposed to appropriate socialization, experiences, and training. Similarly, the PDSA [161] reported that animals newly acquired during lockdown showed behavioral problems relating to a lack of early socialization. Newly acquired animals who were not registered with a vet showed greater signs of aggression and reactivity than newly acquired animals who were registered with a vet, suggesting that perhaps caregivers not registered with a vet lack access to advice about helping their animals adjust [161]. Many younger dogs purchased during the pandemic developed behavioral problems as a result of a lack of socialization and training [113]. Sacchettino et al. [170] also found a significant increase in personality traits related to fear and aggression in young dogs who experienced lockdown during their socialization period, suggesting that pandemic restrictions impacted the behavioral development of young dogs.

Four studies considered the well-being of companion animals acquired during the pandemic, three of which compared pandemic data to pre-pandemic data. There were some negative findings: puppies purchased during the pandemic were less likely to have had veterinary checks before being taken home and less likely to have received all vaccinations (Brand et al., 2022). People who purchased puppies during the pandemic were less likely to see information relating to the health testing of the puppy’s parents or veterinary screening tests (Packer et al., 2021). ‘Pandemic puppies’ were more likely to have skin disorders and parasite infestations [89]. Unsurprisingly, given the restrictions, pandemic puppies were also significantly less likely to have been left alone, socialized, met visitors to the home, met people or dogs from outside the household, walked in a public space, or attended in-person training [89]. Although there was no comparison to puppies purchased pre-pandemic, Wriedt [188] reported that many puppies born during the pandemic found socializing difficult, were scared of other dogs, and had separation anxiety. However, there were also positive findings: people who acquired dogs during the pandemic were more likely to have been given advice on diet, health, exercise, and training [89] and more likely to carry out pre-purchase research [157]. By 2021, however, people were less likely than in 2020 to carry out pre-purchase research [158], although this may be attributable to the fact that many considered themselves experienced and felt they did not need to carry out research. Sherlock et al. [174] found that dogs acquired during the pandemic visited the vet more frequently and were dewormed more frequently; the authors suggested that people who acquired their dogs during lockdown may be more vigilant because they had established stronger relationships with their dogs due to spending more time with them than usual due to social restrictions. 

Finally, the 2021 PDSA report [161] found that people who acquired their animals after March 2020 were significantly more likely to say that their animals made them stressed than those who acquired animals pre-March 2020. The authors suggested this may indicate that ‘pandemic purchases’ were not fully thought-out or prepared for.

#### 3.6.2. The Transition out of Lockdown

One study [106] explored caregivers’ preparation for the transition from lockdown to post-lockdown. Most people reported doing nothing to prepare their animals for this transition; others had gradually increased time away from home, had made efforts to maintain normal routines throughout lockdown, had gradually returned to pre-lockdown routines, or had chosen to continue working from home some of the time in order to not disrupt their animals’ routines. Participants also described gradual resocialization with other animals and people, gradual reintroduction of dogs to doggy daycare, and enriching the home environment with toys and interactive treat feeders. However, the same study found that animal well-being was higher during lockdown than post-lockdown. Additionally, Bennett et al. [79] found that participants who had started returning to work reported the transition was difficult for both themselves and their animals, and they described feelings of anxiety and guilt around leaving their animals alone again.

#### 3.6.3. Interactions with Non-Companion Animals 

One study examined the association between mental health and contact with nature and wildlife (e.g., wild birds, bats, foxes, squirrels) [177]. In this study, such contact was perceived to have a positive impact on humans’ mental health; participants reported awe and privilege when seeing animals in nature and felt this provided an opportunity for distraction from pandemic-related distress. Seeing animals in nature was described as a joy and a comfort that helped people feel less alone, became an important part of the daily routine, and motivated people to learn more about animals. 

#### 3.6.4. Grief and Loss

One study examined predictors of grief following animal loss during the pandemic [150]. Attachment to the animal and experience of other losses during the pandemic predicted greater levels of grief, while isolation was not associated with grief. The study also found, unexpectedly, that greater perceived social support showed an indirect effect on grief through stronger animal attachment. Overall, greater attachment was associated with more intense grief, but attachment was not intensified through loss of social support or increased isolation.

#### 3.6.5. Domestic Abuse 

Hawkins et al. [118] interviewed domestic abuse helpline workers, finding that animal-related concerns raised by callers were not perceived to differ during lockdown to pre-lockdown; some felt the frequency of such calls had increased, but pointed out that the frequency of calls in general (not just animal-related) had increased. Animals were often harmed or used as tools for abuse during lockdown and prevented their caregivers from leaving as they worried about the safety of their animals; however, such was also the case pre-lockdown.

#### 3.6.6. Animal-Focused Well-Being Interventions 

One study [152] involved the evaluation of two animal-focused interventions during lockdown, examining the effect of the interventions on both animals (specifically dogs) and their caregivers. One intervention involved mindfulness, with tasks such as touching dogs’ fur, watching dogs breathe, and mentally tracing an outline of the dog in the mind. The other intervention involved a series of different interactions caregivers must do with their dogs, such as playing ‘hide and seek’, following the dog’s lead, outside interactive play, seven minutes of affection time, taking a selfie with the dog, and talking or reading to the dog. Both interventions brought about a range of positive effects for the caregivers (including feeling more relaxed and connected to their dog), whereas, for the animals themselves, the interaction intervention was perceived to benefit their moods more than the mindfulness intervention. In particular, dogs appeared to enjoy ‘hide and seek’, ‘follow the lead’, and ‘interactive play’. The mindfulness task relating to the dog’s fur was perceived to be relaxing and enjoyable for the dogs. The interaction task relating to taking a selfie together appeared to make dogs restless, while some caregivers felt uncomfortable being instructed to talk to their dogs. Despite caregivers’ perceptions that the interventions helped them to feel more connected to their dogs, neither emotional closeness nor loneliness were statistically associated with the intervention.

#### 3.6.7. Suggestions in Case of Future Pandemics

Applebaum et al.’s [77] participants suggested that in the case of a future pandemic, animal caregivers should be prepared in advance and ensure they have both a stock of supplies such as food and medicine and plans for what would happen to their animals should their caregivers became sick. They also suggested that keeping a routine and seeking community resources for assistance with vet bills and food would be useful. Online (e.g., Zoom) training and online shows and classes gave participants something to do; virtual social activities with other animal caregivers were also found to be helpful and so would be recommended if people were to enter into another lockdown [184]. Wu et al.’s [189] participants suggested that it needed to be easier to access information about financial assistance programs to help with veterinary care and that other service agencies (e.g., mental health clinics) could collaborate with veterinary clinics to increase the visibility of assistance programs.

#### 3.6.8. Relinquishment of Animals

Despite the reported challenges of living with companion animals during the pandemic, the vast majority of participants would not consider giving up their animals [89,102,120,144,154,165]. Of the very few people who would consider rehoming their animals, this tended to be due to financial difficulties in accessing veterinary care, unmanageable behavioral problems and toileting in the house [102], or impaired quality of life of the caregiver [144]. Hoffman et al. [120] found that those who acquired animals during the pandemic were more likely to relinquish them than those whose companion animals had been with them since before the pandemic. Additionally, they found that those who worked from home were more likely to consider rehoming their animals. In the same study, males, older adults, Black (compared to White) participants, those with higher incomes, and those living in urban areas were more likely to have relinquished an animal, and males, younger adults, those with children in the home, those with higher incomes and those in rural or urban areas (compared to suburban) were more likely to be considering relinquishing an animal in the future. 

## 4. Discussion

In this scoping review, we reviewed 122 studies and found positive, negative, and neutral associations between animal companionship and caregiver well-being, as well as numerous self-reported benefits of being an animal caregiver during the pandemic; a multitude of challenges and a number of (caregiver-reported) positive and negative effects on companion animals themselves. 

Similar to previous reviews [24,45,46], we found a mix of positive, negative, and neutral findings regarding the association between animal companionship and mental health/well-being, particularly with regard to depression, anxiety, stress, and loneliness. We also found mixed (positive, negative, and null) evidence of an association between animal companionship and overall mental health/well-being and positive affect. Most other outcomes were only investigated in very few studies, and therefore, no strong conclusions could be drawn about these. 

Although our findings were clearly extremely mixed, we did find more evidence of null associations between animal companionship and well-being than positive or negative associations: in total, twenty-five studies reported at least some non-significant associations. Twenty-one studies reported at least one positive, significant association between animal companionship and well-being (i.e., suggesting that animal companionship benefited well-being). Fewer studies (*n* = 10) reported at least one negative association (i.e., suggesting that animal companionship negatively affected well-being), and in 2/10 studies, these associations lost significance in multivariate analysis. This highlights the importance of considering potential confounding variables in the relationship between animal caregiving and well-being. For example, Denis-Robichaud et al. [101] pointed out that, without adjusting for other variables, their results would have given the impression that people with companion animals had poorer well-being than people without, when, in fact, this was not the case. Rather, people with companion animals were more likely to be female, less educated, lacking a social network, and have disabilities—all characteristics that are also suggested to be risk factors for poor mental health [101]. Analysis of factors affecting the association between animal companionship and well-being yielded similarly inconsistent results.

Overall, our findings echo the fragmented evidence of the relationship between animal companionship and caregiver well-being, which can be seen in pre-COVID literature. The contradictions within our reviewed studies may be due to a number of reasons, such as the variety of research designs used [37]; the number of different variables measured; over-reliance on questionnaires developed specifically for the studies rather than standardized measures; the lack of prospective studies; potentially differing levels of quality across the studies; different values placed on companion animals by different people [20]; socio-demographic factors; and socio-cultural differences between studies carried out in different parts of the world. 

Descriptive qualitative data revealed many perceived benefits of animal guardianship during the pandemic. Interestingly, qualitative data showed that many participants appeared to believe that their companion animals reduced their stress and improved their mental health despite the quantitative evidence relating to stress and mental health being inconclusive. Similar findings have been reported in a previous review in the field, perhaps due to the outcome measures used in quantitative studies not reflecting the impacts that are most important to participants [216] or qualitative data being able to pick up on more nuances and complexities. 

Animals were perceived to benefit mental health in a number of ways during the pandemic. They were seen as helping to provide routine and a sense of structure: this is important as the loss of one’s usual routine during pandemic-related restrictions can be distressing [4], whereas maintaining everyday routines can facilitate well-being [217]. Companion animals gave their caregivers a sense of purpose during lockdown; previous research has suggested that having a purpose (i.e., something that contributes to the world beyond oneself) can be a meaningful resource during the pandemic [218]. Animals were also reported to be a distraction from the stress, fear, and uncertainty participants felt regarding COVID-19. Indeed, too much thinking about the pandemic has the potential to be maladaptive and is associated with a number of negative health outcomes [219], whereas positive distractions may contribute to better well-being [220]. Companion animals have previously been found to provide important distractions from upsetting experiences and symptoms in people with mental health conditions [221]. Animals were also reported to bring joy and entertainment to their caregivers and were perceived to be a calming presence, helping people to relax, reducing sadness, and preventing them from feeling overwhelmed. Additionally, animals were perceived to be a grounding presence, providing a sense of perspective during the crisis and encouraging their caregivers to live in the moment. Enjoyment of the ‘little joys’ in life and learning to live in the moment have been reported to be helpful ways of coping with the pandemic [217,222]. 

A range of psychosocial benefits were also reported. Companion animals were reported to provide a valuable alternative to human connection and a substitute for physical touch from other humans. This connection was undoubtedly important during a time when contact with people outside the home was so restricted. Research has shown that physical touch has beneficial effects on psychological well-being and that ‘longing for touch’ during the pandemic was associated with poorer physical, psychological, and social quality of life [223], so being able to physically touch animals may have been beneficial and calming [224]. Animals were also reported to provide emotional support, comfort, love, a sense of safety, and diminished feelings of loneliness. Similar benefits of companion animals were reported in a previous review of animal companionship for people with mental health problems [24]. In the current review, it was also reported that animals often encouraged interactions with other people during the pandemic—for example, dog walkers greeting other dog walkers, animals being conversation starters during virtual meetings for people working or studying remotely, or caregivers feeling encouraged to reach out and support other animal caregivers. This finding provides support for the suggestion from pre-COVID literature that companion animals can be a conduit for getting to know other people and experiencing social support [225]. Interestingly, despite the quantitative data revealing very mixed results around loneliness and animal companionship, ‘reduced loneliness’ was identified as a theme within qualitative data, but ‘increased loneliness’ was not. The data also suggested that companion animals may be perceived as benefiting well-being more by people who lived alone. 

Another benefit of animal companionship for their caregivers during the pandemic was increased exercise and fresh air, particularly for people with dogs. This echoes some pre-COVID findings [226] but contradicts others, which showed no association between animal companionship and exercise [44]. The quantitative data we reviewed also showed either positive or no associations associations between animal companionship and exercise. Exercise has been reported to help people cope with lockdown during the pandemic [217] to improve their quality of life and reduce depressive symptoms, anxiety, and stress [21]. Other benefits were reported, which related to remote working, with participants suggesting that because of their animals, they took more breaks from the computer and had better work–life balance. This is an important finding, given that remote working appears to be here to stay [227] and that emerging literature on working from home suggests that remote working blurs the distinction between ‘work’ and ‘home’ and can make it difficult to maintain an appropriate work–life balance [228]. 

Participants in many studies reported that relationships with companion animals had improved in terms of increased emotional bonds. Several studies noted that animals’ ability to read caregivers’ body language had improved, and they appeared more attuned to the emotions and needs of their caregivers due to increased time spent together. A pre-COVID review of research on animal companionship for people with mental ill-health also suggested that animals tended to respond to their caregivers intuitively, particularly during times of crisis [24]. Several studies in this review also suggested that lockdowns had been used to develop children’s relationships with animals, helping them to enhance their caring skills and better understand the boundaries of animals. While it is important for children to understand how to appropriately interact with animals in the home—and indeed, companion animals can be beneficial for children’s well-being [229], parents should also ensure this is performed in a safe way that is enjoyable for both the child and the animal. Given that emotions are potentially heightened during lockdown for both people and animals [4,59] and that research suggests an increase in children being injured by animals during lockdown [29], parents should be particularly careful. 

Positively for animals, many studies reported that caregivers perceived their animals’ well-being to have improved during the pandemic. Notably, many animals were perceived to be happier and calmer and reportedly enjoyed the increased companionship from their caregivers being at home so much more. Many were reported to be more playful and more affectionate. For many, exercise had increased, and walks were often more pleasant for dogs and their caregivers due to fewer people and less traffic around. Future research should aim to better understand factors associated with positive changes in animals during crises in order to develop recommendations for caregivers to optimize their animals’ health and well-being.

Four studies described positive aspects of veterinary telemedicine, which was often perceived to be less stressful for both caregivers and animals, as well as quicker, cheaper, and more convenient. However, others struggled with telemedicine and reported many challenges, including delays in diagnoses or treatment, concerns about misdiagnoses, and difficulties communicating with veterinarians. These findings echo reports of both the benefits and challenges of telehealth for humans during the pandemic [230]. Training of veterinarians in effective communication skills for telehealth consultations may be beneficial. Video consultations might also be more useful than telephone consultations, enabling veterinary staff to observe the animals on screen: this might help communication and potentially aid the diagnostic process. 

Various other challenges of being an animal caregiver during the pandemic were reported. Many of these related to concerns about meeting companion animals’ needs: in particular, participants appeared to be concerned about reduced access to veterinary care and difficulties procuring animal food, medication, and other supplies. Similar fears have been reported around difficulties accessing medical care [231] and food for humans during the pandemic [232]. Having inadequate supplies during confinement may not only be a source of frustration during confinement but could continue to be associated with poorer well-being afterward [4]. Therefore, animal food banks might be a useful resource for caregivers during health crises [5]. Additionally, participants reported challenges meeting the social and behavioral needs of their animals, describing concerns that animals were not getting enough stimulation or enrichment during lockdown and were missing out on training and socializing. This led to concerns about behavioral problems developing or worsening. Spending quality time with animals where they have their caregivers’ full attention may be useful, and for many animals, the provision of enrichment activities and problem-solving toys might provide mental stimulation for them while they are cooped up in the house. 

Many participants reported concerns relating to COVID-19 infection. Most commonly, participants feared what would happen to their animals if they were to become ill or hospitalized with COVID-19. Similar concerns have been reported about children in other studies; people quarantined with COVID-19 described fears over what would happen to their children [233]. Importantly, some participants in our reviewed studies reported that they would delay COVID-19 testing or treatment due to concerns about what would happen to their animals. Many studies have examined factors associated with intentions to test for COVID-19 [234,235,236], but most do not consider the role of companion animals in that decision. We did not find many studies that looked at animals as a predictor of COVID-19 testing or treatment (*n* = 3), but those that did found a substantial number of participants would indeed delay testing and treatment due to concerns about what would happen to their animals should they be hospitalized; all found that at least some participants would delay testing or treatment (including 10% of participants in one study and 65% of participants in another). This finding is of major public health importance as it suggests companion animals might play a role in caregivers’ adherence to COVID-19 testing. Additionally, delay in treatment-seeking could be very detrimental to health. These findings somewhat echo those of companion animal studies of natural disasters, which have found that concern for animals’ well-being can result in people returning to high-risk areas or refusing to evacuate [55,237,238,239,240] or planning to take their animals with them even if this could affect their own safety [241]. Even outside of disasters, companion animal caregivers have reported that they would delay hospitalization for illness because of their animals [242]. 

Other COVID-19-related concerns included concerns about animals catching COVID-19 themselves or transmitting the virus to humans and concerns about infection risk when doing activities relating to animals, such as shopping for supplies, seeking veterinary care, or walking dogs. Fears of infection are common during pandemics and can cause substantial distress [4]. Early in the COVID-19 pandemic, inconsistent media reports and a lack of clarity around whether animals could catch and spread the virus would have compounded these fears [66]. 

Numerous other challenges were reported, including animals distracting their caregivers from work; balancing different caregiving roles; concerns about animals picking up on caregivers’ stress; fears about how animals would cope when caregivers returned to work, and financial concerns. Financial concerns are common during confinement and can be a risk factor for developing psychological disorders [4]. Another psychological challenge for caregivers was the emotional difficulty of having to wait outside while their animals saw veterinarians; they wanted to be with them to support them and hold them during appointments and found it distressing to be separated from them. In future public health crises, policymakers should consider allowing caregivers to be present for appointments as long as infection control guidelines are properly adhered to. 

Some negative impacts of the pandemic on companion animals were reported, although these tended to be reported by only a minority of participants. The most commonly reported changes were increased attention-seeking and clinginess. We examined predictors of negative behavioral/well-being impacts for animals but found little relevant data and inconsistent findings. There was some evidence that animals risked negative behavioral or well-being changes if they lived in crowded households or their caregivers had poor quality of life; for dogs, inadequate exercise appeared to predict negative changes. Animals newly acquired during lockdown appeared to be particularly at risk for negative behaviors, presumably due to a lack of training and socialization caused by the restrictions of the pandemic. Concerningly, we found some evidence that people purchased or adopted new animals to improve the lockdown experience, which suggests they may not have had the animals’ best interests at heart, given that the lockdown was only ever going to be temporary. We also found some evidence that transitioning out of lockdown was difficult for both animals and their caregivers. 

A small number of studies examined animal loss during the pandemic. Some participants who had experienced this described the heartbreak of not being able to be with their animals in their last moments due to pandemic restrictions; others feared experiencing this. Those who did lose animals tended to find that grieving was more difficult during the pandemic because they could not rely on their usual social support networks. Research on the loss of (human) loved ones during lockdown has shown similar findings, with bereavement during the pandemic potentially leading to prolonged grief due to the difficulties of mourning alone [243]. Additionally, research on the loss of companion animals during natural disasters has suggested that losing an animal during a disaster is associated with greater post-traumatic stress disorder [244,245], distress [246], and depression [245]. 

Due to climate change, urbanization, and accessibility of travel, pandemics are likely to increase in both intensity and frequency [247,248]. It is, therefore, important to consider the findings of this review in terms of what they mean in the case of future pandemics or other similar prolonged crises. 

### 4.1. Implications for People Considering Purchasing/Adopting New Animals during a Crisis

Evidence suggests that adoptions and purchases of animals soared early in lockdown [16]. However, we suggest that decisions around bringing new animals into the home—especially for people who have not been animal caregivers before—should be well thought-out and take into account strains on personal and financial resources [74]. Given the many challenges caregivers faced during the pandemic and the reduced access to training and socialization, which negatively affected young animals, a crisis with social restrictions such as a pandemic may not always be the optimum time to bring new animals into the family. For people who are prepared, understand the challenges, and will still have time to spend with their animal(s) in everyday life when restrictions have eased, it may be appropriate and even beneficial for both humans and animals to bring an animal into the home. However, for many others, lockdown is unlikely to be the best time to acquire new animals. 

For those for whom it would not be appropriate to have a companion animal, there are other options. For example, this review found that interacting with nature and being able to see wildlife (where possible, depending on public health guidelines) can be beneficial for well-being—and without the challenges of animal caregiving. Additionally, emerging research suggests potential positive impacts of interactions with robot pets. For example, robot companion pets have been shown to decrease depression and loneliness in older adults with dementia during the pandemic [249] and significantly decrease neuropsychiatric symptoms in older adults in care homes [250]. A scoping review of nine studies revealed robot pets could have positive impacts on mood and affect, communication, companionship, and well-being [251]. People who might benefit from these, such as older people, appear to be open to engaging with robotic pets and participating in robotic pet programs [252]. Additionally, virtual reality animals could be a promising alternative that could reduce stress and induce positive emotions [253,254]. 

### 4.2. Implications for Existing Animal Caregivers

Using the findings of this review, we suggest that the following recommendations might benefit companion animals and their caregivers in the case of another pandemic or similar prolonged crisis with restrictions on movement and socialization. 

*Outside of a crisis it may be worth considering what would be needed if a crisis were to occur:*

- Ensure appropriate stocks of supplies are kept in the home, including animal food and medication where possible;
- Keep on top of vaccines and healthcare treatment for animals throughout the year so there is no rush to seek them *during* the crisis;
- Develop a plan for who would care for animals (e.g., family, friends, neighbors, temporary foster care) in the event of illness, hospitalization, or inability to care for animals due to workload [5].

*During a pandemic/other prolonged crisis:*

- Maintain a routine and structure as much as possible; this is likely to benefit both caregivers and animals [209];
- Exercise and fresh air (while adhering to public health guidelines) are also likely to benefit both caregivers and animals;
- Be careful when allowing children and animals to spend time together, being wary of the fact that both children and animals might be bored and frustrated and should be separated if either is showing negative behaviors toward the other;
- Seek up-to-date information (e.g., about the risk of animals catching or transmitting infections) from appropriate, evidence-based sources such as peer-reviewed literature, the World Health Organization, or government reports, as opposed to relying on the media or social media for information;
- Space-permitting, if required to work remotely, set up a dedicated ‘working from home’ space away from the distraction of companion animals. This could reduce the risk of distractions and also provide much-needed relaxation time for the animals;
- Develop a plan for transitioning back to ‘normal life’, considering how this will affect animals and how to mitigate any risks;
- Continue any ongoing training for animals; this could be performed remotely, over Zoom, or individually based on guidance from evidence-based sources;
- For animals such as cats and dogs, who may be allowed to roam freely within the home, they may benefit from a designated ‘hiding place’ or safe space they can go to when they do not wish to be disturbed. Such a space should be dark, soundproofed, and easily accessible for them but away from family activity [209];
- Enrichment activities and mental stimulation, especially those that do not involve humans [209], may benefit animals. For example, puzzle feeders and problem-solving toys may be helpful.

### 4.3. Implications for Policy-Makers

- It is important, during pandemics and any disasters, to consider animals in emergency preparedness and response efforts—not only because it is important for the animals themselves but because, if animals are not considered properly, this could lead to substantial preventable mental health problems given that a substantial proportion of the population have a companion animal;
- Disaster planning may necessitate coordination between emergency management and animal welfare agencies [255];
- Setting up animal food banks and food bank delivery services would be beneficial [5] both for the animals themselves and the mental health of the animal caregiver population;
- Ensure information about any financial support available (e.g., for animal food or veterinary care) is easily accessible to caregivers; for example, advertising support services in animal magazines, in supermarkets, and in veterinary surgeries.

### 4.4. Implications for Future Research

We identified some research gaps where further research is needed.

- Overall, further research is needed to elucidate the impact of companion animals on caregiver well-being during the pandemic; more longitudinal, prospective studies of the general population during the COVID-19 pandemic, adjusting for a variety of potentially confounding variables, would help further our understanding of the complex relationship between animal caregiving and well-being. In particular, longitudinal, prospective studies could help us to understand whether animal caregivers are more likely to experience the negative impacts of the pandemic or whether those with poorer life satisfaction are more likely to have animals in the first place;
- As most studies were cross-sectional in nature and had collected data early in the pandemic, few studies were able to describe the transition out of lockdown and how this might affect companion animals and their guardians. More research on this transition is needed;
- More research on the relatively unexplored area of how concerns about companion animals influence virus testing or healthcare treatment is needed;
- More research on animal loss during a crisis is needed, in particular, exploring ways of supporting people who were not allowed to be with their animals during end-of-life care;
- In future health emergencies, policy-makers should allow people to be with their animals during end-of-life care, as the limitations in place during COVID-19 could have had profound effects on both the animals in their last moments and the grieving process of their caregivers. The importance of being with dying (human) relatives has been established, and during the pandemic, scholars argued for increased access to dying loved ones despite the risk of infection [256]. We suggest the same should be true for animals if appropriate guidelines are adhered to, such as observing proper infection control procedures [256];
- Further research on alternatives to companion animals (e.g., spending time in nature, robot pets, virtual reality animal interactions) is necessary to understand how the well-being of people who are not able to bring a companion animal into their home might be benefited.

### 4.5. Limitations 

There are a number of limitations to the reporting of both the reviewed literature and the review itself. In terms of the literature, we found a lack of longitudinal and prospective studies, with the vast majority providing cross-sectional data, which means that while associations could be explored, causation cannot be assumed. It has been suggested that people with and without companion animals may differ in socio-demographic characteristics and that the tendency for animal caregivers to be from advantaged and majority backgrounds may inflate the positive association between guardianship and well-being [74]. Prospective longitudinal research would help to elucidate this. 

Few studies focused on companion animals who were not cats or dogs, meaning our findings may not be as relevant to other animal caregivers as to dog or cat caregivers. Additionally, few studies assessed when animals were brought into the home—if they were acquired in lockdown, they might be more likely to display negative behaviors and potentially cause additional stress to their caregivers due to lack of training and socialization. 

Regarding the review itself, firstly, our decision to limit the review to studies published in the English language potentially affects the findings. Opting to translate studies published in other languages may have yielded many more studies. We noted a lack of studies from Asia and particularly from Africa were lacking in this review, which may be in part due to our language limitation. Secondly, only one author carried out the screening, data extraction, and data synthesis; ideally, studies would be double-screened and data extracted in duplicate in order to ensure reliability. Additionally, as this was a scoping study rather than a systematic review, we did not carry out a quality appraisal or risk of bias assessment of the included studies. This would have benefited the review as, particularly where findings are conflicting, this might have provided some insight as to whether the inconsistencies were between higher-quality studies and poorer-quality studies. Finally, as this was a scoping review, our aim was to provide a broad, summarized synthesis of all the key concepts emerging from this large body of research. As such, we were unable to focus on smaller details and nuances within the research. For example, we recorded the benefits and challenges of animal companionship reported in each study, but we did not record the percentage of participants who reported each benefit or challenge. This could be important in terms of quantifying the benefits and challenges experienced and providing a more detailed overall picture of how companion animals affected the lockdown experience. Additionally, we note that the time period in which studies were conducted is important (i.e., whether data were collected during strict lockdown or after restrictions had begun to ease), and while we recorded this data in Supplementary Table S1, we did not separate the results by study date as this was considered beyond the scope of the current review. Similarly, we did not attempt to separate data by type of companion animal or examine data from specific sub-groups separately. As one of the purposes of a scoping review is to indicate whether more specific systematic reviews with a narrower focus might be needed [69], we suggest that should future researchers plan to systematically review pandemic-related data relating to animal companionship; they should consider how study quality, number of participants reporting each outcome, time period, companion animal species, and specific caregiver population might influence the findings. 

## 5. Conclusions

Overall, the findings of this review highlight the considerable inconsistencies in the literature relating to the effect of companion animals on caregiver well-being during a time of unique crisis, change, and uncertainty. Positive, negative, and null findings were reported, making it difficult to gain a true understanding of how being an animal caregiver might affect individuals’ pandemic experiences. Qualitative findings tell us that there are many potential benefits of animal companionship during a pandemic, with animals providing a daily routine, a sense of purpose, positive distraction, companionship, emotional support, and a reason to interact with other humans. Animals themselves can benefit from the extra companionship, and human–animal bonds can increase. However, there are also many challenges, such as concerns about access to food and veterinary care, concerns about meeting animals’ social and behavioral needs, fears around virus transmission, financial worries, and the risk of avoiding virus testing or treatment due to worries about what would happen to animals if their caregivers were hospitalized. Further longitudinal, prospective, and high-quality research studies are needed to fully understand the association between animal companionship and caregiver well-being and to untangle the effects of the pandemic on both caregivers and their companion animals. However, it is clear that caregivers feel that their animals have provided companionship, love, and emotional support during the pandemic, and it is important that love and support are reciprocated by considering the well-being of animals both as the world returns to ‘normal’ and in the planning for future pandemics or other prolonged crises.

## Figures and Tables

**Table 1:** Inclusion and exclusion criteria.

Inclusion Criteria	Exclusion Criteria
Primary data	Reviews; theoretical/conceptual papers with no new data collected
Studies examining the impact of the COVID-19 pandemic on companion animal caregiver and/or companion animal well-being, either through statistical analysis or qualitative descriptions of challenges and benefits experienced during the pandemic	Studies not considering well-being specifically in relation to the COVID-19 pandemic
Participants must be companion animal caregivers reporting on their own well-being, animal caregivers reporting on their animals’ well-being, or other key stakeholders (e.g., veterinary staff) reporting on caregivers’ or animals’ well-being; other human non-caregivers were included only if their well-being data were compared to that of animal caregivers	Studies consisting only of participants who are not animal companions, key stakeholders, or involved in the care of animals
Research study with 2+ participants	Case studies; auto-ethnographic papers which did not involve research

**Table 2:** Statistical associations between being a companion animal caregiver and psychological well-being.

Well-Being Outcome	Positive Findings	Negative Findings	No Association with Animal Companionship
**Anxiety**	Lower anxiety: Gasteiger et al. [109]; Giansanti et al. [110]	Greater anxiety: Clements et al. [97] * ; Denis-Robichaud et al. [101] * ; Law et al. [134]	Gijón Puerta et al. [111]; Grajfoner et al. [112]; Hawkins and Brodie [116]; Lima et al. [136] (dogs only); Martin et al. [138]; Shah et al. [173]
**Depression**	Lower depression: Bohn et al. [85] (dogs only); Gasteiger et al. [109]; Martin et al. [138]	Greater depression: Clements et al. [97] *; Law et al. [134]	Bohn et al. [85] (cats and birds only); Gijón Puerta et al. [111]; Grajfoner et al. [112]; Hawkins and Brodie [116]; Lima et al. [136] (dogs only); Shah et al. [173]; Wells et al. [185]
**Distress**	Lower distress: Damberg and Frömbling [100]	-	-
**Stress**	-	Greater stress: Denis-Robichaud et al. [101] *; Mueller et al. [147]; Ogata et al. [151] (cats only)	Gijón Puerta et al. [111]; Grajfoner et al. [112]; Hawkins and Brodie [116]; Ogata et al. [151]; Shah et al. [173]; Wells et al. [185]
**Tension-anxiety**	Namekata and Yamamoto [149] (dogs only)	-	-
**Post-traumatic growth**	Higher post-traumatic growth: Dominick [103]	-	Dominick et al. [104]
**Overall mental health**	Smaller decrease in mental health scores: Ratschen et al. [165]	Poorer mental health: Denis-Robichaud et al. [101] *; Greater decline in mental health: Shoesmith et al. [178]	-
**Overall general health**	-	Poorer health: Denis-Robichaud et al. [101] *	Tan et al. [179]
**Physical health**	-	-	Shoesmith et al. [178]
**Quality of life**	-	Poorer quality of life: Denis-Robichaud et al. [101] *; Phillipou et al. [162]	Oliver-Hall et al. [154]
**Life satisfaction**	-	Lower satisfaction: Amiot et al. [74]	
**General well-being**	Greater well-being: Damberg and Frömbling [100]; Grajfoner et al. [112]	Lower well-being: Amiot et al. [74]	Barklam and Felisberti [78]; Hawkins and Brodie [116]; Kuehne et al. [132]
**Emotional well-being**	Greater well-being: Sánchez-Ferrer et al. [171]; Tan et al. [179]	-	-
**Positive emotions/positive affect**	Greater positive emotions: Grajfoner et al. [112]; Junça-Silva et al. [125]	Lower positive affect: Mueller et al. [147] (non-dog animals only)	Hoffman [119]; Martinez-Caja et al. [139]; Mueller et al. [147] (dogs only); Wells et al. [185]
**Happiness**	-	-	Martin et al. [138]
**Optimism**	-	-	Barklam and Felisberti [78]
**Negative emotions/negative affect**	-	-	Grajfoner et al. [112]; Hawkins and Brodie [116]; Hoffman [119]; Martinez-Caja et al. [139]
**Self-efficacy**	-	-	Oliver-Hall et al. [154]
**Coping self-efficacy**	Higher coping self-efficacy: Grajfoner et al. [112]	-	-
**Healthy coping behaviors**	Higher odds of healthy coping: Mueller et al. [147] (dogs only) *	-	Mueller et al. [147] (non-dog pets only)
**Mindfulness**	-	Lower mindfulness: Oliva and Johnston [153] (cats only)	Oliva and Johnston [153] (dogs only)
**Presence of life meaning**	-	Amiot et al. [74]	-
**Resilience**	-	-	Barklam and Felisberti [78]; Grajfoner et al. [112]; Phillipou et al. [162]
**Disruption of core beliefs**	-	-	Dominick et al. [104]
**Isolation**	Lower isolation: Damberg and Frömbling [100]; Hart et al. [114] (dogs only); van der Velpen et al. [180]	-	-
**Loneliness**	Lower loneliness: Lau and Oliva [133]; Martinez-Caja et al. [139]; Oliva and Johnston [153] (dogs only); van der Velpen et al. [180] Smaller increase in loneliness during lockdown: Ratschen et al. [165]	Greater loneliness: Amiot et al. [74]; Denis-Robichaud et al. [101] *; Mueller et al. [146]	Barklam and Felisberti [78]; Dost et al. [105]; Gasteiger et al. [109]; Oliva and Johnston [153] (cats only); Phillipou et al. [162]; Wells et al. [185]
**Emotional loneliness caused by deficits in family relationships**	-	-	Ogata et al. [151]
**Emotional loneliness caused by deficits in romantic relationships**	Lower emotional loneliness: Ogata et al. [151]	-	-
**Social loneliness resulting from lack of friendships or workplace relationships**	-	-	Ogata et al. [151]
**Socializing**	Increased socializing: Hoffman [119] (dogs only)	-	-
**Social functioning**	Greater social functioning: Tan et al. [179]	-	-
**Social connectedness**	-	-	Kuehne et al. [132]; van der Velpen et al. [180]
**Satisfaction with social roles**	-	-	Oliver-Hall et al. [154]
**Likelihood of spending time with family or participating in sports as a coping strategy**	-	Less likely to cope by spending time with family or exercising: Mueller et al. [146]	-
**Perceived social support**	Greater support: Martin et al. [138]	-	Dominick et al. [104]
**Energy**	Greater energy: Tan et al. [179]	-	-
**Vitality**	-	-	Amiot et al. [74]
**Effects of remote working**	Greater perceived positive effects: Junça-Silva et al. [125]	-	-
**Job performance**	Greater perceived performance: Junça-Silva et al. [125]	-	-
**Amount of physical activity**	More activity: Hoffman [119] (dogs only); Mueller et al. [147] (dogs only; higher odds of having a walking routine); Tan et al. [179] (low-intensity activity only)	-	Gasteiger et al. [109]; Mueller et al. [147] (non-dog pets only, no association with having a healthy walking routine); Tan et al. [179] (moderate or vigorous activity only)
**Time spent outdoors in fresh air**	More time outside: Moore et al. [143] (dogs only); Mueller et al. [147] (all pets in univariate analysis, dogs only in multivariate analysis)	-	-
**COVID-19 impacts**	-	Higher COVID-related impacts: Amiot et al. [74]	-
**Coronavirus anxiety**	-	-	Kuehne et al. [132]
**Perceived difficulties of the pandemic**	-	-	Namekata and Yamamoto [149]
**Basic psychological need satisfaction (autonomy, competence, relatedness)**	-	-	Barklam and Felisberti [78]

* = univariate association only; lost significance in multivariate regression.

**Table 3:** Perceived benefits of having a companion animal during the COVID-19 pandemic for humans, and benefits of the pandemic for animals.

Finding	Evidence
***Psychological benefits for humans***	
Companion animals perceived to reduce caregivers’ stress, tension and distress	Adams et al. [73]; Bennetts et al. [81]; Bolstad et al. [86]; Bussolari et al. [90]; Currin-McCulloch et al. [98]; Flores-Flores et al. [108]; Jezierski et al. [122]; Jezierski et al. [123]; Kirnan et al. [126]; Koochaknejad et al. [130]; Krouzecky et al. [131]; Mueller et al. [146]; Namekata and Yamamoto [149]; Shoesmith et al. [177]; Victor and Mayer [181]; Wriedt [188]; Zablan et al. [192]
Companion animals perceived to improve caregivers’ mental health and well-being	Barklam and Felisberti [78]; Bussolari et al. [90]; Clements et al. [97]; Currin-McCulloch et al. [98]; Dogs Trust [102]; Holland et al. [121]; Kogan et al. [127]; Oliver-Hall et al. [154]; Owczarczak-Garstecka et al. [156]; Shoesmith et al. [177]; Shoesmith et al. [178]; Victor and Mayer [181]
Provided a semblance of normality and stability by allowing humans to maintain a structure and routine	Adams et al. [73]; Applebaum et al. [77]; Bennett et al. [79]; Bennetts et al. [81]; Bolstad et al. [86]; Bussolari et al. [90]; Clements et al. [97]; Currin-McCulloch et al. [98]; Dogs Trust [102]; Holland et al. [121]; Kirnan et al. [126]; Kogan et al. [127]; Oliva and Johnston [153]; Owczarczak-Garstecka et al. [156]; Reniers et al. [166]; Shoesmith et al. [177]; Shoesmith et al. [178]; Victor and Mayer [181]; Ward et al. [184]; Zablan et al. [192]
Gave life a purpose and meaning, preventing feelings of uselessness	Bennett et al. [79]; Bennetts et al. [81]; Bussolari et al. [90]; Clements et al. [97]; Currin-McCulloch et al. [98]; Dogs Trust [102]; Holland et al. [121]; Kirnan et al. [126]; Kogan et al. [127]; Oliva and Johnston [153]; Owczarczak-Garstecka et al. [156]; Scholtz [172]; Shoesmith et al. [178]; Zablan et al. [192]
Animals displaced worry and served as a distraction, reprieve, and something positive to focus on	Adams et al. [73]; Applebaum et al. [77]; Bennetts et al. [81]; Bussolari et al. [90]; Clements et al. [97]; Currin-McCulloch et al. [98]; Holland et al. [121]; Kirnan et al. [126]; Owczarczak-Garstecka et al. [156]; Reniers et al. [166]; Shoesmith et al. [177]; Victor and Mayer [181]; Zablan et al. [192]
Animals helped caregivers to cope emotionally during a time of uncertainty	Bennett et al. [79]; Bennetts et al. [81]; Clements et al. [97]; Currin-McCulloch et al. [98]; Johnson and Volsche [124]; Kogan et al. [127]; Mueller et al. [146]; Oliver-Hall et al. [154]; Zablan et al. [192]
Animals provided joyful, pleasant, cosy feelings and made the home a positive environment	Adams et al. [73]; Bennett et al. [79]; Flores-Flores et al. [108]; Reniers et al. [166]; Ribeiro et al. [167]; Scholtz [172]; Victor and Mayer [181]; Wriedt [188]
Animals diminished feelings of being overwhelmed	Kogan et al. [127]; Scholtz [172]
Animals helped caregivers to relax	Dogs Trust [102]; Krouzecky et al. [131]; Ribeiro et al. [167]
Animals helped children and youth in the family to feel good	Caldwell et al. [91]; Zainel et al. [193]
Animals were a calming presence	Bussolari et al. [90]; Currin-McCulloch et al. [98]; Kirnan et al. [126]; Krouzecky et al. [131]; Lukoševičiūtè and Šmigelskas [137]; Pawar et al. [159]; Scholtz [172]; Shoesmith et al. [177]; Shoesmith et al. [178]; Victor and Mayer [181]
Animals reduced the sadness of being separated from family	Flores-Flores et al. [108]
Animals provided a sense of perspective	Clements et al. [97]; Owczarczak-Garstecka et al. [156]
Animals helped caregivers to feel more grounded	Currin-McCulloch et al. [98]
Animals provided a reminder to live in the moment	Owczarczak-Garstecka et al. [156]
Animals improved mood	Applebaum et al. [77]; Bennet et al. [79]; Dogs Trust [102]; Holland et al. [121]; Oliva and Johnston [153]
Animals were a source of fun, entertainment, and laughter	Bennetts et al. [81]; Currin-McCulloch et al. [98]; Dogs Trust [102]; Lukoševičiūtè and Šmigelskas [137]; Oliva and Johnston [153]; Owczarczak-Garstecka et al. [156]; Shoesmith et al. [178]
Animals perceived to improve self-compassion	Kogan et al. [127]
Animals fostered a sense of gratitude	Bussolari et al. [90]; Currin-McCulloch et al. [98]
Animals boosted morale	Charmaraman et al. [95]
***Psychosocial benefits for humans***	
Animals provided companionship, an alternative to human interpersonal connections	Applebaum et al. [77]; Bennetts et al. [81]; Bussolari et al. [90]; Charmaraman et al. [95]; Clements et al. [97]; Dogs Trust [102]; Oliva and Johnston [153]; Owczarczak-Garstecka et al. [156]; Shoesmith et al. [177]; Shoesmith et al. [178]; Victor and Mayer [181]; Ward et al. [184]; Zablan et al. [192]
Animals provided psychological/emotional support	Applebaum et al. [77]; Bennett et al. [79]; Clements et al. [97]; Reniers et al. [166]; Scholtz [172]; Shoesmith et al. [177]; Victor and Mayer [181] ; Ward et al. [184]
Animals provided a source of comfort and love	Adams et al. [73]; Barklam and Felisberti [78]; Bennett et al. [79]; Bennetts et al. [81]; Bussolari et al. [90]; Clements et al. [97]; Currin-McCulloch et al. [98]; Holland et al. [121]; Kirnan et al. [126]; Krouzecky et al. [131]; Martinez-Caja et al. [140]; Owczarczak-Garstecka et al. [156]; Scholtz [172]; Shoesmith et al. [177]; Shoesmith et al. [178]; Victor and Mayer [181]; Wriedt [188]; Zablan et al. [192]
Animals provided a comforting substitute for human touch/physical contact	Adams et al. [73]; Applebaum et al. [77]; Bussolari et al. [90]; Clements et al. [97]; Oliva and Johnston [153]; Owczarczak-Garstecka et al. [156]; Shoesmith et al. [177]; Victor and Mayer [181]
Animals diminished feelings of isolation and loneliness	Adams et al. [73]; Bussolari et al. [90]; Currin-McCulloch et al. [98]; Holland et al. [121]; Kirnan et al. [126]; Kogan et al. [127]; Oliva and Johnston [153]; Owczarczak-Garstecka et al. [156]; Reniers et al. [166]; Scholtz [172]; Shoesmith et al. [177]; Shoesmith et al. [178]; Wriedt [188]; Zablan et al. [192]
Animals provided a sense of safety, security, and protection	Reniers et al. [166]; Scholtz [172]; Victor and Mayer [181]
Animals encouraged interpersonal connections with other humans and were often a conversation starter	Bennett et al. [79]; Bennetts et al. [81]; Clements et al. [97]; Dogs Trust [102]; Holland et al. [121]; Oliva and Johnston [153]; Owczarczak-Garstecka et al. [156]; Reniers et al. [166]; Scholtz [172]; Zablan et al. [192]
Caregivers felt encouraged to reach out and support other animal caregivers (e.g., sharing advice online)	Wu et al. [190]
Pleasure/emotional regulation derived from providing care	Flores-Flores et al. [108]; Zablan et al. [192]
***Health-related benefits for humans***	
Animals helped people to increase exercise, stay fit, and spend time outdoors in fresh air, green spaces, and nature	Applebaum et al. [77]; Bennetts et al. [81]; Bolstad et al. [86]; Bussolari et al. [90]; Dogs Trust [102]; Esam et al. [106]; Holland et al. [121]; Krouzecky et al. [131]; Moore et al. [143]; Oliva and Johnston [153]; Oliver-Hall et al. [154]; Owczarczak-Garstecka et al. [156]; Reniers et al. [166]; Shoesmith et al. [177]; Shoesmith et al. [178]; Ward et al. [184]; Zablan et al. [192]
***Work-related benefits for humans***	
Animals encouraged caregivers to take breaks from their computers	Dogs Trust [102]; Owczarczak-Garstecka et al. [156]; Scholtz [172]; Victor and Mayer [181]
Animals encouraged better work–life balance	Bolstad et al. [86]
Animals perceived to reduce work stress	Scholtz [172]
Animals perceived to improve productivity	Scholtz [172]
Animals increased motivation	Scholtz [172]
***Other benefits for humans***	
Financial concerns encouraged caregivers to find out about financial support available	Wu et al. [190]
***Benefits for human–animal relationships***	
Increased emotional bonds between caregivers and companion animals	Bowen et al. [87]; Bowen et al. [88]; Bussolari et al. [90]; Currin-McCulloch et al. [98]; Denis-Robichaud et al. [101]; Dogs Trust [102]; Kogan et al. [127]; Kogan et al. [128]; Kogan et al. [129]; Lee et al. [135]; Martinez-Caja et al. [140]; Oliva and Johnston [153]; Riggio et al. [168]
Increased companionship, interactions and quality time spent together	Adams et al. [73]; Barklam and Felisberti [78]; Bolstad et al. [86]; Bowen et al. [87]; Bowen et al. [88]; Bussolari et al. [90]; Christley et al. [96]; Currin-McCulloch et al. [98]; Dogs Trust [102]; Kogan et al. [128]; Kogan et al. [129]; Lee et al. [135]; Oliva and Johnston [153]; Ribeiro et al. [167]; Riggio et al. [168]; Shoesmith et al. [176]; Wu et al. [190]
Enhanced intimacy due to ability of human and animal to read each other’s body language	Bussolari et al. [90]; Shoesmith et al. [177]; Victor and Mayer [181]; Wu et al. [190]; Zablan et al. [192]
Perceived psychological costs/challenges of having a companion animal were reduced	Bowen et al. [88]; D’Angelo et al. [99]
Increased appreciation for companion animals	Shoesmith et al. [177]; Shoesmith et al. [178]
Lockdown provided time for children to take more responsibility for animals/develop their caring skills	Adams et al. [73]; Zainel et al. [193]
Lockdown allowed children to better understand animals’ boundaries	Adams et al. [73]
***Benefits for animals’ well-being***	
Animals perceived to be happier	Bussolari et al. [90]; Esam et al. [106]; Kirnan et al. [126]; Oliva and Johnston [153]; Pawar et al. [159]
Reduced anxiety when about to be left alone	Dogs Trust [102]
Animals enjoyed the increased company	Boardman and Farnworth [84]; Esam et al. [106]; Holland et al. [121]
More stimulation	Esam et al. [106]
Animals perceived to be calmer and more relaxed	Boardman and Farnworth [84]; Bowen et al. [87]; Dogs Trust [102]; Esam et al. [106]; Jezierski et al. [122]; Jezierski et al. [123]; Morgan et al. [144]; Oliva and Johnston [153]; Riggio et al. [168]; Shoesmith et al. [176]
Animals perceived to be more playful	Jezierski et al. [122]; Jezierski et al. [123]; Shoesmith et al. [176]
Animals perceived to be more affectionate	Bussolari et al. [90]; Esam et al. [106]; Martinez-Caja et al. [140]; Shoesmith et al. [176]
Animals perceived to be less stressed	Platto et al. [164] (exception of cats)
Behavioral problems reduced	Platto et al. [164]
Decreased coughing in animals	Woolley et al. [187]
Animals getting more exercise	Boardman and Farnworth [84]; Dogs Trust [102]; Esam et al. [106]; Lee et al. [135]; Oliva and Johnston [153]; Woolley et al. [187]
Decreased reactivity	Boardman and Farnworth [84]
More opportunities for training	Dogs Trust [102]; Esam et al. [106]; Shoesmith et al. [176]
More pleasant walks for reactive dogs with less people around	Dogs Trust [102]; Owczarczak-Garstecka et al. [156]
Safer to be outside as less traffic around	Esam et al. [106]
Aquariums better maintained than previously and had more money spent on them	Koochaknejad et al. [130]
***Advantages of veterinary telemedicine***	
Less stressful for animals	Caney et al. [93]
Less stressful for caregivers	Caney et al. [93]
Quicker assessments	Caney et al. [93]
Avoiding transportation and time in waiting room	Caney et al. [93]
Convenience	Caney et al. [93]
Safer	Caney et al. [93]
Appreciation of veterinarian’s communication regarding COVID transmission risks and extra safety precautions taken	Kogan et al. [128]; Kogan et al. [129]
Reduced cost	Caney et al. [93]

**Table 4:** Perceived challenges of having a companion animal during the COVID-19 pandemic for humans and challenges of the pandemic for animals.

Finding	Evidence
***Concerns about meeting animals’ basic needs***	
Concerns about/difficulties procuring food for animals	Applebaum et al. [75]; Applebaum et al. [77]; Bennett et al. [79]; Bussolari et al. [90]; Currin-McCulloch et al. [98]; Dogs Trust [102]; Oliver-Hall et al. [154]; Owczarczak-Garstecka et al. [156]; Ratschen et al. [165]; Rombach and Dean [169]
Concerns about/difficulties procuring other animal care supplies (e.g., cat litter, toys, leashes, beds, bowls)	Applebaum et al. [75]; Applebaum et al. [77]; Bennett et al. [79]; Bennetts et al. [81]; Clements et al. [97]; Esam et al. [106]; Oliver-Hall et al. [154]; Owczarczak-Garstecka et al. [156]; Shoesmith et al. [176]
Concerns about other people panic buying/hoarding supplies	Applebaum et al. [75]; Applebaum et al. [77]; Bennett et al. [79]; Currin-McCulloch et al. [98]; Owczarczak-Garstecka et al. [156]
Concerns about/difficulties procuring medication	Applebaum et al. [75]; Bowen et al. [87]; Owczarczak-Garstecka et al. [156]
Concerns about/difficulties accessing veterinary care/appointments	Applebaum et al. [75]; Applebaum et al. [77]; Bennett et al. [79]; Bennetts et al. [81]; Bolstad et al. [86]; Bowen et al. [87]; Bussolari et al. [90]; Clements et al. [97]; Currin-McCulloch et al. [98]; Dogs Trust [102]; Esam et al. [106]; Holland et al. [121]; Jezierski et al. [122]; Jezierski et al. [123]; Kirnan et al. [126]; Kogan et al. [128]; Kogan et al. [129]; Morris et al. [145]; Oliver-Hall et al. [154]; Owczarczak-Garstecka et al. [156]; Ratschen et al. [165]; Shoesmith et al. [177]; Shoesmith et al. [178]; Ward et al. [184]; Williams et al. [186]; Wriedt [188]; Wu et al. [190]
No access to professional grooming services	Applebaum et al. [75]; Clements et al. [97]; Owczarczak-Garstecka et al. [156]
Difficulties walking and exercising animals	Bennetts et al. [81]; Bowen et al. [87]; Clements et al. [97]; Esam et al. [106]; Holland et al. [121]; Krouzecky et al. [131]; Martinez-Caja et al. [140]; Oliver-Hall et al. [154]; Ratschen et al. [165]; Shoesmith et al. [176]
***Concerns about meeting animals’ social and behavioral needs***	
Concerns about animals not getting enough enrichment/stimulation	Applebaum et al. [75]; Bussolari et al. [90]; Dogs Trust [102]; Holland et al. [121]
Animals missing out on physical touch	Bussolari et al. [90]
Animals missing out on day-care	Applebaum et al. [75]; Bussolari et al. [90]; Owczarczak-Garstecka et al. [156]
Animals missing out on socialization	Applebaum et al. [75]; Applebaum et al. [77]; Bennetts et al. [81]; Bussolari et al. [90]; Dogs Trust [102]; Holland et al. [121]; Martinez-Caja et al. [140]; Owczarczak-Garstecka et al. [156]
Dogs missing out on service dog/therapy dog activities	Bussolari et al. [90]; Shoesmith et al. [176]
Dogs missing out on dog sports and play activities	D’Angelo et al. [99]
Loss of professional dog-walkers	Owczarczak-Garstecka et al. [156]
Animals missing out on training	Applebaum et al. [75]; Bennetts et al. [81]; Dogs Trust [102]; Martinez-Caja et al. [140]; Owczarczak-Garstecka et al. [156]
Lack of control over animals’ routines	Ward et al. [184]
Concerns about animals developing behavioral issues	Bussolari et al. [90]; Holland et al. [121]
Concerns about animals’ chronic behavioral problems worsening	Applebaum et al. [75]
Concerns about needing to retrain animals in future	Holland et al. [121]
Difficulties balancing adherence with public health guidelines and meeting animals’ needs	Owczarczak-Garstecka et al. [156]
***COVID-related concerns***	
Concerns about what would happen to animals if caregivers were ill, incapacitated, or hospitalized	Applebaum et al. [75]; Applebaum et al. [76]; Applebaum et al. [77]; Bennett et al. [79]; Bennetts et al. [81]; Bolstad et al. [86]; Bussolari et al. [90]; Currin-McCulloch et al. [98]; Dogs Trust [102]; Holland et al. [121]; Kogan et al. [128]; Kogan et al. [129]; Krouzecky et al. [131]; Oliver-Hall et al. [154]; Ratschen et al. [165]; Williams et al. [186]
Humans likely to delay or avoid testing or treatment for COVID-19 due to concerns about what would happen to their animals	Applebaum et al. [76]; Dogs Trust [102]; Matijczak et al. [141]
Worries about animals catching COVID-19	Applebaum et al. [75]; Bennett et al. [79]; Bennetts et al. [81]; Bolstad et al. [86]; Bussolari et al. [90]; Clements et al. [97]; Currin-McCulloch et al. [98]; Dogs Trust [102]; Esam et al. [106]; Shoesmith et al. [177]; Williams et al. [186]
Worries about humans catching COVID-19 from animals	Applebaum et al. [75]; Bolstad et al. [86]; Clements et al. [97]; Dogs Trust [102]; Esam et al. [106]; Oliver-Hall et al. [154]; Shoesmith et al. [177]; Williams et al. [186]
Fear of having to euthanize animals if they caught COVID-19	Currin-McCulloch et al. [98]
Concerns about potential infection risks involved in exercising animals, seeking veterinary care, or shopping for animal supplies	Applebaum et al. [75]; Bolstad et al. [86]; Dogs Trust [102]; Holland et al. [121]; Morris et al. [145]; Owczarczak-Garstecka et al. [156]; Wu et al. [189]
Concerns about needing to physically distance from animals if caregivers developed COVID-19	Applebaum et al. [75]
Exhausting to look after animals when suffering Long-COVID	Krouzecky et al. [131]
***Challenges of remote working/studying with companion animals in the home***	
Animals demanding attention when working from home	Applebaum et al. [75]; Applebaum et al. [77]; Bennett et al. [79]; Bussolari et al. [90]; Scholtz [172]; Victor and Mayer [181]
Animals interrupting/being vocal during video conferences	Applebaum et al. [75]; Bennetts et al. [81]; Bolstad et al. [86]; Scholtz [172]; Victor and Mayer [181]
Animals distracting humans from work	Applebaum et al. [75]; Hoffman [119]; Kirnan et al. [126]; Scholtz [172]; Victor and Mayer [181]
Concerns about animals interrupting children studying from home	Bennetts et al. [81]
***Psychological challenges for humans***	
Irritation, frustration and annoyance at animals	Applebaum et al. [75]; Krouzecky et al. [131]
Guilt around being at home but not able to give animal full attention	Bussolari et al. [90]
Reduced mental health for those who were separated from animals (e.g., horses kept elsewhere)	Williams et al. [186]
Balancing competing demands of animal care and other caregiving responsibilities, home-schooling or work	Applebaum et al. [75]; Bennetts et al. [81]; Bussolari et al. [90]; Kirnan et al. [126]; Scholtz [172]
Fear of own anxieties exacerbating animals’ anxiety/animals picking up on stress	Bussolari et al. [90]; Martinez-Caja et al. [140]
Concerns about animals developing separation anxiety/not coping when caregivers return to work/restrictions lifted	Applebaum et al. [75]; Bennetts et al. [81]; Boardman and Farnworth [84]; Bolstad et al. [86]; Bussolari et al. [90]; Dogs Trust [102]; Esam et al. [106]; Hoffman et al. [120]; Holland et al. [121]; Oliva and Johnston [153]; Oliver-Hall et al. [154]; Owczarczak-Garstecka et al. [156]; Ratschen et al. [165]; Shoesmith et al. [177]; Shoesmith et al. [178]; Zablan et al. [192]
Emotional challenges of having to wait curbside during veterinary appointments	Bennett et al. [79]; Dogs Trust [102]; Gregory [113]; Kogan et al. [128]; Kogan et al. [129]; Morris et al. [145]; Wu et al. [189]; Wu et al. [190]
Financial concerns, e.g., less money to spend on animals, concerns about being able to afford to care for animals if furloughed	Applebaum et al. [75]; Applebaum et al. [77]; Bennett et al. [79]; Bennetts et al. [81]; Esam et al. [106]; Hoffman et al. [120]; Kirnan et al. [126]; Kogan et al. [128]; Kogan et al. [129]; Morris et al. [145]; Oliver-Hall et al. [154]; PDSA [161]; Shoesmith et al. [177]; Williams et al. [186]; Wu et al. [189]
Loss of interaction with other animal caregivers	Ward et al. [184]
Concerns about other people’s companion animals being affected by increased domestic abuse during lockdown	Esam et al. [106]
General concerns about other animals after the pandemic (e.g., people abandoning their animals after COVID restrictions eased, separation anxiety, reduced exercise, boredom)	Bennetts et al. [81]; Dogs Trust [102]; Esam et al. [106]; Shoesmith et al. [177]
Children could be jealous of animals preferring adult caregivers	Adams et al. [73]
COVID limited the ability to knock on doors/conduct thorough searches if animal went missing	Bennetts et al. [81]
***Health-related challenges for humans***	
Allergies to animal dander exacerbated due to spending more time at home	Bennetts et al. [81]
***Negative impacts on animal behavior***	
Increased neediness, attention-seeking, insecurity or clinginess	Applebaum et al. [75]; Applebaum et al. [77]; Bennett et al. [79]; Bennetts et al. [81]; Boardman and Farnworth [84]; Bowen et al. [87]; Currin-McCulloch et al. [98]; Dogs Trust [102]; Esam et al. [106]; Harvey et al. [115]; Holland et al. [121]; Martinez-Caja et al. [140]; Morgan et al. [144]; Oliva and Johnston [153]; Owczarczak-Garstecka et al. [156]; Ribeiro et al. [167]; Riggio et al. [168]; Scholtz [172]; Sherwell et al. [175]; Shoesmith et al. [176]; Shoesmith et al. [177]
Increased or excessive vocalization	Bowen et al. [87]; D’Angelo et al. [99]; Dogs Trust [102]; Esam et al. [106]; Owczarczak-Garstecka et al. [156]; Platto et al. [164]; Ribeiro et al. [167]; Sherwell et al. [175]
Increased nervousness, shyness or fears (e.g., of loud noises)	Boardman and Farnworth [84]; Bowen et al. [87]; Dogs Trust [102]; Esam et al. [106]; Kirnan et al. [126]; Owczarczak-Garstecka et al. [156]; Ribeiro et al. [167]; Sacchettino et al. [170]; Shoesmith et al. [176]
Separation anxiety	Bennett et al. [79]; Bennetts et al. [81]; Blue Cross [83]; Boardman and Farnworth [84]; Esam et al. [106]; Harvey et al. [115]; Holland et al. [121]; Kirnan et al. [126]; PDSA [160]; PDSA [161]; Scholtz [172]; Sherwell et al. [175]; Wriedt [188]
Various behaviors relating to lack of socialization (e.g., signs of fear, aggression, nervousness)	PDSA [161]
‘Unsettled and anxious’	Bennetts et al. [82]
Increased excitability	Boardman and Farnworth [84]; Bowen et al. [87]
Increased frustration	Bowen et al. [87]; Dogs Trust [102]
Increased agitation	Bolstad et al. [86]
Increased restlessness	Holland et al. [121]; Owczarczak-Garstecka et al. [156]
Increased reactivity	Boardman and Farnworth [84]; Owczarczak-Garstecka et al. [156]
Increased hyperactivity	Ribeiro et al. [167]
Increased stress	Bennetts et al. [81]; Bowen et al. [87]; Esam et al. [106]; Platto et al. [164] (cats only)
Increased anxiety	Boardman and Farnworth [84]; Shoesmith et al. [176]
Increased irritability	Bowen et al. [87]; Shoesmith et al. [176]
Increased aggression	Boardman and Farnworth [84]; Platto et al. [164]; Sacchettino et al. [170]; Sherwell et al. [175]
Increased destructive behavior	Holland et al. [121]; PDSA [160]; Ribeiro et al. [167]
Increased mouthing/nipping	Holland et al. [121]
Increased territoriality	Kirnan et al. [126]
Increased toileting accidents in the home	Esam et al. [106]; Ribeiro et al. [167]
Less social	Boardman and Farnworth [84]; Kirnan et al. [126]
Expectations of increased attention	Applebaum et al. [75]
Training regression	Boardman and Farnworth [84]
More behaviors associated with stress during veterinary appointments	Caney et al. [92]; Muzzatti and Grieve [148]
General behavioral issues due to lack of socialization and training	Gregory [113]
***Negative impacts on animal health and well-being***	
Changes in appetite	Bolstad et al. [86]; Jezierski et al. [122]
Less exercise	Christley et al. [96]; Owczarczak-Garstecka et al. [156]; Platto et al. [164]; Shoesmith et al. [176]; Vučinić et al. [182]
Weight gain/over-feeding	Bennetts et al. [81]; Esam et al. [106]; PDSA [160]; PDSA [161]; Ribeiro et al. [167]; Shoesmith et al. [176]
Increased health issues including diarrhoea, skin problems, constipation, decreased mobility	Jezierski et al. [122]; Jezierski et al. [123]
Interrupted sleep and relaxation	Bolstad et al. [86]; Esam et al. [106]
Increased noise in the house	Esam et al. [106]
Children initiating unwanted interactions with animals	Adams et al. [73]
Less time spent with caregivers (due to increased working hours or lack of access to horses kept elsewhere)	Shoesmith et al. [177]; Williams et al. [186]
Dogs had fewer interactions with other dogs	Christley et al. [96]; Dogs Trust [102]; Esam et al. [106]; Oliva and Johnston [153]; Owczarczak-Garstecka et al. [156]; Ribeiro et al. [167]; Shoesmith et al. [176]; Wriedt [188]
Dogs restricted to leashes on walks	Esam et al. [106]
Lack of car sense due to not being around cars	Esam et al. [106]
Disrupted routines	Bennett et al. [79]; Bennetts et al. [81]; Esam et al. [106]; Pawar et al. [159]; Williams et al. [186]
Less variety in walks	Owczarczak-Garstecka et al. [156]
***Challenges of veterinary telemedicine***	
Lack of clinical examination	Caney et al. [93]
Delays in receiving diagnosis or treatment	Caney et al. [93]
Risk of misdiagnosis	Caney et al. [93]; Gregory [113]
Difficulties communicating with vets	Caney et al. [93]; Kogan et al. [128]; Kogan [129]; Morris et al. [145]; Wu et al. [189]
Perceived to be stressful	Caney et al. [93]
Price quotations not always clear, resulting in discrepancies between true costs and what participants believed they would be paying	Wu et al. [189]
Veterinarians overwhelmed due to adoption blitzes leading to surge in demand for care	Muzzatti and Grieve [148]
***Animal loss***	
Restrictions in end-of-life care, e.g., not being allowed to be present for euthanasia	Applebaum et al. [75]; Bennetts et al. [81]; Blue Cross [83]; Kogan et al. [128]; Owczarczak-Garstecka et al. [156]
More time at home and the events of 2020 prompted reflection on mortality of animals and heightened fears of loss	Bennetts et al. [81]
Fear of, or having to, grieve alone if animal needed to be euthanised	Applebaum et al. [75]; Shoesmith et al. [177]
More time at home made the grieving process more difficult, exacerbating feelings of loss and impacting mental health	Bennetts et al. [81]

## Data Availability

Not applicable.

## Supplementary Materials

The following supporting information can be downloaded at: https://www.mdpi.com/article/10.3390/ani13203294/s1, File S1: PRISMA-ScR Checklist designed by Tricco et al. [71]; File S2, Table S1: Characteristics of included studies.

## Appendix A

**Search strategy**

1. COVID*
2. coronavirus
3. lockdown*
4. pandemic*
5. stay-at-home
6. shelter-in-place
7. quarantine
8. self-isolat*
9. 1 or 2 or 3 or 4 or 5 or 6 or 7 or 8
10. “dog”
11. dogs
12. “cat”
13. cats
14. “pet”
15. pets
16. animal*
17. 10 or 11 or 12 or 13 or 14 or 15 or 16
18. “mental health”
19. well-being
20. well-being
21. psychological
22. psychiatric
23. depression
24. depressive
25. depressed
26. anxious
27. anxiety
28. ptsd
29. trauma*
30. stress*
31. distress*
32. resilien*
33. coping
34. disorder*
35. mood*
36. happiness
37. sadness
38. sleep*
39. “post-traumatic growth”
40. “posttraumatic growth”
41. “substance abuse”
42. “substance misuse”
43. “substance use”
44. “hazardous drinking”
45. “alcohol use”
46. “alcohol abuse”
47. “alcohol misuse”
48. alcoholi*
49. sleep
50. insomnia
51. loneliness
52. 18 or 19 or 20 or 21 or 22 or 23 or 24 or 25 or 26 or 27 or 28 or 29 or 30 or 31 or 32 or 33 or 34 or 35 or 36 or 37 or 38 or 39 or 40 or 41 or 42 or 43 or 44 or 45 or 46 or 47 or 48 or 49 or 50 or 51
53. 9 and 17 and 51
54. Limit to 2019 or later
55. Limit to English language

## Appendix B

**Studies excluded after full-text screening**

*Studies found* via *database searching*

Chiu et al. [195]: No data on animal companionship (focus on acceptance of animals and robots as companions)
Forward et al. [196]: No data on animal companionship
Gu et al. [197]: Animal companionship not entered as a potential predictor variable
Han [198]: Not a research study (auto-ethnographic commentary)
Hunjan and Reddy [7]: No primary data
Ikeuchi et al. [199]: Data collected pre-pandemic
Kogan et al. [200]: No COVID-specific data
Mayers [201]: Not a research study (auto-ethnographic commentary)
McMillan et al. [202]: No individual-level data
Oliva and Johnston [203]: No COVID-specific data
Passavanti et al. [204]: No data on animals
Ramesh et al. [205]: No primary data
Wong et al. [206]: No COVID-specific data

*Studies found* via *hand-searching*

Arluke et al. [207]: Unclear if data collected during the pandemic
Aydemir et al. [208]: No COVID-specific data
Hargrave [209]: No primary data
Hargrave [210]: No primary data
Hockenhull et al. [211]: No well-being data
Hui Gan et al. [212]: Data collected pre-pandemic
Jalongo et al. [213]: No primary data
King [214]: No primary data
