# Supplementary material for: The Well-Being of Companion Animal Caregivers and Their Companion Animals during the COVID-19 Pandemic: Scoping Review
Source: Animals (Basel). 2023 Oct 22;13(20):3294. doi: 10.3390/ani13203294 (PMC10604194; doi:10.3390/ani13203294)
Supplement: Supplementary file 1 [file animals-13-03294-s001.zip › Supplementary File S2.pdf]

Supplementary Table S1. Characteristics of included studies

Authors (year)	Country	Study population	Socio-demographic characteristics	Type of pet	Study design	Human-related aspects assessed	Animal-related aspects assessed	Time period of data collection
Adams et al. (2021) [73]	United States of America	2,537 adult pet owners	89.1% female<br><br>Mean age 39.3 (range 18 – 85)	Not reported	Cross-sectional survey	-	What would happen to pets if owners hospitalized for COVID-19; open-ended question about how presence of pet affected children during COVID-19	April – May 2020
Amiot et al. (2022) [74]	Canada	2,424: 1,220 pet owners vs. 1,204 non-pet owners	49.5% male, 50.2% female, 0.3% other<br><br>Approximately 50% were aged 50+	Various	Cross-sectional survey	Extent of stress as a result of COVID-19; extent of uncertainty as a result of COVID-19; extent of family-related issues as a result of COVID-19; vitality; loneliness; life satisfaction; stress	'Do you have one or more pet(s) currently?'; 'How many pets do you currently have?'; species of pets	September – October 2020
Applebaum et al. (2020a) [75]	United States of America	2,254 pet owners	89% female<br><br>Mean age 39, range 18-85	Various	Cross-sectional survey with open-ended responses	-	Attachment to pets; if and how pets add stress to the COVID-19 situation; difficulties with pets due to social distancing or shelter-in-place; pros and cons of living with pets	April – July 2020

							during the pandemic	
Applebaum et al. (2020b) [76]	United States of America	1,356 pet owners	89.2% female<br><br>Mean age 39.0 (range 18-82)	Not reported	Cross-sectional survey with open-ended questions	Factors associated with healthcare decision-making	Attachment to pets; would participants delay or avoid COVID-19 testing or treatment because of worries about pet's welfare	April – May 2020
Applebaum et al. (2021) [77]	United States of America	2,068 pet owners	89.5% female, 7.8% male, 2.8% other<br><br>Mean age 39.6	Not reported	Cross-sectional survey with open-ended responses	Social support; loneliness	Attachment to pets; pros and cons of living with pets during the pandemic; advice for people living with pets in future pandemics	April – July 2020
Barklam & Felisberti (2023) [78]	Authors in United Kingdom; Study 1 had an international sample, Study 2 had a UK sample	495 (Study 1 – 344 pet owners vs. 151 non-pet owners)<br>243 (Study 2)	Study 1: Pet owners – 78% female, 22% male; Non-pet owners – 67% female, 33% male<br><br>Mean age 31.66, range 18-73<br><br>Study 2: Pet owners – 75% female, 25% male; Non-pet owners – 74% female, 26% male	Dogs, cats, other	Two cross-sectional surveys	Loneliness; wellbeing; resilience; optimism; basic psychological need satisfaction	How pandemic affected routines and interactions with pets, and ability to care for pet; pet attachment	Study 1: May - June 2020<br>Study 2: September 2021

			Mean age 27.7, range 18-38					
Bennett et al. (2022) [79]	Australia	250 adults with companion animals	212 females, 34 males, 4 non-binary	Various	Cross-sectional survey with open-ended questions	Satisfaction with life; mental wellbeing	Pet dependency; perceived emotional support from pets; pets' effect on emotional experiences	June – August 2020
Bennetts et al. (2022a) [80]	Australia	1,034 parents living with a child under 18 and a cat or dog	77.7% female, 21.8% male, 0.1% non-binary, 0.5% prefer not to say<br><br>Mean age 43, range 20-65	Cats, dogs	Cross-sectional survey	107 closed-end questions and 2 open-ended items including parent and child wellbeing, child anxiety, COVID-19 impact	Human-pet attachment	July - October 2020
Bennetts et al. (2022b) [81]	Australia	611 parents with a child under 18 and a cat or dog	80.7% female, 18.6% male, 0.2% non-binary, 0.5% prefer not to say<br><br>Mean age 43.0	Cats, dogs	Open-ended questions on a cross-sectional survey	-	Challenges and benefits of having a cat or dog during pandemic	July – October 2020
Bennetts et al. (2023) [82]	Australia	1,034 parents living with a child under 18 and a cat or dog	78% female<br><br>Mean age 43, range 20-65	Cats, dogs	Cross-sectional survey	Parent and child psychological wellbeing	Benefits of having a pet; worries relating to the pet; engagement in pet-related activities	July – October 2020
Blue Cross (n.d.) [83]	United Kingdom	2,946 pet owners	Not reported	Dogs, cats, small animals	Cross-sectional survey	-	Pet-related experiences during the pandemic	May – June 2020

Boardman & Farnworth (2022) [84]	United Kingdom (England)	15 dog owners	9 females, 6 males<br><br>Age not reported	Dogs	Qualitative interviews	Work situation during COVID-19	Dogs' social behavior during and after lockdown	December 2021 – January 2022
Bohn et al. (2021) [85]	Brazil	1,123 older adults	90.7% female<br><br>Mean age 67.68	Dogs, cats, birds	Cross-sectional survey	Depression	Pet ownership – yes/no; type of pet	June 2020
Bolstad et al. (2021) [86]	United States of America	102 dog or cat owners	91.2% female<br><br>Mean age 38 (range 21-84)	Dogs, cats	Cross-sectional survey with open-ended questions	-	If a new pet was acquired during pandemic; engagement with pet during the pandemic and in the month prior (time spent with pet, responsibilities, time spent physically touching pets, pleasantness of engaging with pet, upset feelings toward pets, attachment to pets); how COVID-19 experience was affected by being a pet owner; if pet was helpful or unhelpful in coping with social distancing; concerns relating to pet during pandemic	April – May 2020

Bowen et al. (2020) [87]	Spain	1,297 adult pet owners (794 dog owners, 503 cat owners)	90.5% female<br><br>Mean age not reported	Cats, dogs	Cross-sectional survey	Socio-demographics; type of home; effect of confinement; number of weeks confined; negative financial, emotional, health and lifestyle effects of confinement; quality of life	Number of pet cats and dogs; cat/dog-owner relationship scale to assess human-animal bond on three dimensions (interaction, emotional closeness, perceived costs); effect of confinement on pet's quality of life; anger towards pet; changes in pet's behavior; confinement concerns; pre-confinement outdoor access	Not reported, but participants had been in confinement for an average of 3.2 weeks at the point of data collection (lockdown was imposed in March 2020) and the paper was submitted May 2020
Bowen et al. (2021) [88]	Spain	794 dog owners	90.2% female, 9.8% male<br><br>Mean age 40.7	Dogs	Comparative surveys	-	Cat/dog owner relationship	Representative sample data collected early 2019; convenience sample provided data 'two weeks after the start of Spanish lockdown'
Brand et al. (2022) [89]	United Kingdom	4,369 'pandemic puppies' purchased between	Pandemic puppies: owners 90% female	Dogs	Comparison study using surveys	-	Attending puppy classes; puppy health issues; veterinary care	November – December 2020

		March – December 2020 vs. 1,148 '2019 puppies' purchased during 2019	Age group most represented was 25-54 (48.7%)<br><br>2019 puppies: owners 92% female<br><br>Age group most represented was 25-54 (48.6%)					
Bussolari et al. (2021) [90]	Authors in USA; participants global (80.7% USA, 8.7% Canada, 4.3% UK, 1.6% Australia, 4.7% other	4,105 adults with companion dogs	88.3% female, 10.8% male, 0.8% non-binary, 0.4% no answer<br><br>Mean age 48.06 (range 18-92)	Dogs	Cross-sectional survey	Levels of social restriction; access to social support; coping	Human-animal bond; pandemic-related changes in relationships with dogs; stressors; ways in which COVID-19 impacted feelings about dogs; access to dog food and medical care	March - April 2020
Caldwell et al. (2021) [91]	Canada	699 parents of children/youth and 279 youth	Parents: 53% female<br>Age not reported<br><br>Youth: 68.5% cisgender female, 26.2% cisgender male, 1.4% transgender male, 0.4%	Not reported	Cross-sectional survey	Health behaviors (physical activity, sleep, screen time); thoughts and feelings about the pandemic	-	August 2020

			transgender female, 1.1% non-binary, 0.7% two-spirited, 0.4% other, 1.4% prefer not to say<br><br>Age group most represented was Grade 10-12 (68.5%)					
Caney et al. (2022a) [92]	Authors in United Kingdom; participants were international	371 veterinary consultations (210 standard vs. 161 during COVID-19)	Not reported	Cats	Cross-sectional survey	-	Experiences of veterinary appointments; cat behavior and stress during appointment	March – July 2020
Caney et al. (2022b) [93]	Authors in United Kingdom; participants were international	242 veterinary professionals, 98 owners with experience of telemedicine	Not reported	Cats	Cross-sectional survey	-	Perceived advantages and disadvantages of telemedicine for animals	September – December 2020
Carr et al. (2021) [94]	United States of America	466 older adults	66% female<br><br>Mean age 69.4, range 60-92	Dogs	Longitudinal	Loneliness (controlled for other pet ownership, health and financial impact of pandemic, impact of other stressful societal events, socio-demographics, social support);	Frequency of dog-walking	September 2018 and October 2020

						social impact of COVID-19		
Charmaraman et al. (2022) [95]	United States of America	Survey: 567 pet-owning adolescents and subset of 356 dog owning adolescents aged 10-17<br><br>Interviews: 31 parents of adolescents	Pet owning adolescents: 55% female, 44% male, 1% non-binary or missing gender<br><br>Mean age 13.09, range 10-17<br><br>Dog owning adolescents: 55% female, 45% male<br><br>Mean age 13.24, range 10-17<br><br>Parents: 28/31 female, 2/31 male, 1/31 non-binary<br><br>Age not reported	Various	Mixed-methods	Surveys: Coping strategies; family relationships during COVID-19; socio-demographics	Pet ownership – yes/no; dog ownership – yes/no; pet caretaking responsibility; top 5 identities (e.g. friend, athlete, student, pet owner)<br><br>Interviews: how, when and why they acquired pets; coping and stress management; caretaking responsibilities; social media and digital technology as it relates to the pet; child emotional attachment to the pet; impact of COVID on having a pet	Fall 2020
Christley et al. (2021) [96]	United Kingdom	6,004 dog owners	85.7% female, 14.2% male, 0.1% self-identified<br><br>Age not reported	Dogs	Cross-sectional survey	-	Dog behavior; management and environment of the dog; dog-walking practices; interactions with people and other	Data collected May 2020; participants provided data for the 7 days prior to survey completion and

							dogs; enrichment activities	retrospective data for February 2020
Clements et al. (2021) [97]	Authors in United Kingdom; international sample	1,199 adults (quantitative survey), 757 open-ended survey responses 25 interviews	Quantitative survey: 82% female, 17% male, 1% other, 1% prefer not to say<br><br>Age group most represented was 18-34 (38%)<br><br>Qualitative: 88% female<br><br>Age group most represented was 18-34 (40%)	Dogs, cats, fish, small mammals, exotic animals, birds, other	Mixed-methods	Survey: socio-demographics; social and lifestyle behaviors during pandemic; loneliness; mental wellbeing; depression, anxiety and stress; changes in wellbeing during pandemic	Survey: Which type of companion animals; where animal lived/slept; if they were the primary caregiver; interaction affected by pandemic?; influence of animal on wellbeing; level of engagement; accessing veterinary treatment; other pandemic issues regarding animal care; level of concern about contracting/giving COVID from/to animals<br><br>Interviews: Experiences of having a companion animal during the pandemic	Quantitative data: June - November 2020<br><br>Qualitative data: June 2020 - January 2021
Currin-McCulloch et al. (2021) [98]	Authors in United States of America;	956 cat owners	91.5% female, 7.7% male, 0.3% non-	Cats	Cross-sectional qualitative	-	Whether increased time with cat	March 2020 – May 2020

	participants from United States of America (95.7%), Canada (0.4%), United Kingdom (0.8%), Australia (0.2%), other (2.8%)		binary, 0.4% prefer not to answer<br><br>Age group most represented was 50-59 (27%)		survey with open-ended questions		strengthened or strained the relationship; perceived impact of cat on stress; any other thoughts about living with a cat during the pandemic	
D'Angelo et al. (2021) [99]	Italy	2,028 dog owners	81.39% female, 18.61% male<br><br>Age group most represented was 30-40 (24.7%)	Dogs	Cross-sectional survey	-	Information relating to dog and its management, including provenance and behavioral problems; dog-owner relationship scale; dog's lifestyle during lockdown; clinical and behavioral symptoms in dogs; owner perception of distress and emotional discomfort in dogs	May 2020
Damberg & Frömbling (2022) [100]	United States of America	215 pet owners	63.3% male, 36.7% female	Various	Cross-sectional survey	Psychological distress; subjective wellbeing; social	Giving and needing of animal support	October 2020

			Age group most represented was 18-35 (60.5%)			isolation during COVID		
Denis-Robichaud et al. (2022) [101]	Canada	1,500 adults	50.2% female, 49.5% male, 0.3% other or did not answer<br><br>Age group most represented was 45-54 (20.5%)	Various	Cross-sectional survey	Quality of life (including mobility, self-care, usual activities, pain and discomfort, anxiety and depression); loneliness; mental health; wellbeing before and during pandemic<br>Potential confounders – demographics; physical and mental health, current social support, attitude toward companion animals and recent changes in ownership, employment status and change	Presence of at least one companion animal in the household – yes/no	April - May 2021
Dogs Trust (2020) [same dataset as Christley et al., 2020] [102]	United Kingdom	6,004 dogs	Not reported	Dogs	Cross-sectional survey	-	Dogs' behavior /management during last seven days (lockdown) and during February 2020 (pre-lockdown);	May 2020

							owners' bond with dogs; positive and negative aspects of having a dog during lockdown	
Dominick (2022) [103]	United States of America	201 adults	77.6% female<br><br>Mean age 35.39 (range 18-81)	Not reported	Longitudinal	Human social support; post-traumatic growth; coping strategies; stressful events; how COVID-19 challenged core beliefs	Pet ownership – yes/no; if yes – social support provided by pets	T1: March 2020<br>T2: April 2020<br>T3: September 2020<br>T4: March 2021
Dominick et al. (2022) [104]	United States of America	420 adults	80% female<br><br>Mean age 32.51	Not reported	Longitudinal	Human social support; social media use for coping during isolation; post-traumatic growth; degree to which COVID-19 challenged core beliefs	Pet ownership – yes/no; if yes – social support provided by pets	T1: March – April 2020<br>T2: April – May 2020
Dost et al. (2022) [105]	Germany	491 homeless individuals	82.3% male<br><br>Mean age 43.4 (range 18-80)	Various	Cross-sectional survey	Loneliness	Pet ownership – yes/no	July - September 2021
Esam et al. (2021) [106]	New Zealand	T1: 686 pet owners<br><br>T2: 498 pet owners	Age, gender not reported	Dogs, cats, other	Two cross-sectional surveys at different time-points (unknown whether any of the same people	-	Perceived benefits and negative effects of the pandemic on pets	T1: April 2020<br>T2: July 2020

					completed both)			
Falck et al. (2022) [107]	Canada	12,068 adults (4,718 pet owners vs. 7,350 non-owners)	48.9% male<br><br>Mean age 65.01 (range 45-86 at study entry)	Not reported	Longitudinal	Diagnosis of mental health disorder; depression; anxiety	Pet ownership – yes/no	Participants had completed surveys in 2015-2018, then again April - May 2020 and September - December 2020
Flores-Flores et al. (2023) [108]	Peru	40 older adults	60% female, 40% male<br><br>Mean age 72.1	Various	Qualitative interviews	Feelings, thoughts and behaviors relating to economic circumstances, employment, interactions, social support, coping activities and adaptations	-	August - December 2020
Gasteiger et al. (2021) [109]	New Zealand	681 adults	89.3% female, 10% male, 0.6% other, 0.1% prefer not to say<br><br>Mean age 42, range 18-87	Various	Cross-sectional survey	Depression; anxiety; stress; positive mood; worry about contracting COVID; loneliness; perceived risk of catching COVID; engagement in smoking, exercise and alcohol consumption	Pet ownership – yes/no	May - June 2020
Giansanti et al. (2022) [110]	Italy	781 elderly individuals (405 pet owners, 376 without pets)	50.7% male, 49.3% female	Dogs, cats	Cross-sectional survey	Anxiety	Pet ownership (yes/no); number of pets owned; if pet lives in the house; most	Not reported; described to be 'immediately at the

			Mean age 71.7 (males), 71.4 (females)				relaxing aspects of having a pet; sensations associated with pet	beginning of restrictions'
Gijón Puerta et al. (2022) [111]	Spain, Costa Rica	942 university students	82.6% female, 17.4% male<br><br>Mean age 23.8, range 18-58	Not reported	Cross-sectional survey	Anxiety; stress; depression	Pet ownership – yes/no	May 2020
Grajfoner et al. (2021) [112]	Malaysia	448 adults	58.5% female, 41.5% male<br><br>Mean age 34.75, range 18-70	Dogs, cats, birds, other	Cross-sectional survey	Mental wellbeing; depression, anxiety and stress; resilience; coping self-efficacy; positive and negative affect	Pet ownership (yes/no); type of companionship (pets only, pets and humans, humans only, none); type of pet	June - July 2020
Gregory (2023) [113]	United Kingdom	17 (dog owners who purchased dogs during the pandemic and professionals such as veterinarians, animal welfare organization staff and breeders)	Not reported	Dogs	Interviews, document analysis, observational research	-	Interviews assessed how the pandemic impacted the welfare of dogs; document analysis examined how social distancing protocols affected dogs; observations of dog breeders' breeding, raising and selling conditions	Not reported
Hart et al. (2022) [114]	Authors in United States of America; unclear	147 AIDS survivors	79.6% male, 20.4% female	Cats, dogs	Cross-sectional survey (also retrospectively	Sources of support; experiences with stigma, aloneness,	Owning a cat or dog during the AIDS pandemic and/or the	May - July 2021

	where participants were based		Age group most represented was 61-70 (38.1%)		assessing experiences of the AIDS pandemic 1981-1997)	isolation, sadness/grief and social support during the early AIDS pandemic and the COVID-19 pandemic	COVID-19 pandemic; positive and negative consequences associated with having a pet; support provided in caring for the pet	
Harvey et al. (2022) [115]	United Kingdom	1,807 dogs	Not reported	Dogs	Follow-up study	-	Time dog was left alone without company; separation-related behaviors	February 2020 (data collected retrospectively in May – July 2020), October 2020
Hawkins & Brodie (2020) [conference abstract] [116]	Not reported	86 individuals	Not reported	Not reported	Follow-up study	Psychological wellbeing; depression; anxiety; stress; negative affect	Pet ownership – yes/no	Dates not reported; participants completed surveys at three time points, two weeks apart, during lockdown
Hawkins et al. (2022a) [117]	Authors in the United Kingdom; participants in United Kingdom, United States of America, Canada, Brazil, Australia	117 caregivers reporting on children, 77 child self-reports	Caregivers: 91 mothers, 17 fathers, 7 other Age not reported<br><br>Children: 57% female, 43% male Mean age 10 (range 7-13)	Dogs	Cross-sectional survey	Caregiver-reported child behavior toward dog; caregiver-reported child psychopathology; caregiver-reported child emotion regulation	Number of dogs in the house; age, sex and breed of dog; place of dog acquisition; whether child helped choose dog; whether child considered dog to be their own; child-reported	January – August 2021

							attachment to dogs	
Hawkins et al. (2022b) [118]	United Kingdom	11 domestic abuse helpline staff	91% female, 9% male<br><br>Mean age 39.6 (range 24-56)	Not reported	Qualitative interviews	-	Nature and frequency of helpline calls relating to companion animals within the home	January – May 2021
Hoffman (2021) [119]	United States of America	454 teleworkers	231 females<br><br>Mean age 41.3, range 19-72	Dogs, cats	Cross-sectional survey	Experiences of working from home, including interference of work and personal life; positive and negative emotions	Household included dog or cat – yes/no; how likely they were to pet their cat or dog for 5+ consecutive minutes, play with their cat or dog, and walk their dog on days working from home and days in the office; how having a pet factored into experience of working from home and in the office	June 2020
Hoffman et al. (2021) [120]	United States of America	10,044 adults	46.7% male, 53.3% female<br><br>Age group most represented was 55+ (37.8%)	Dogs, cats	Cross-sectional survey	COVID-related worries	Ownership of cats or dogs – yes/no; whether participants planned to rehome an animal in the next 3 months; whether they had acquired	May – June 2021

							or fostered animals during pandemic; whether they had relinquished an animal and reasons for relinquishing animal; animal-related worries	
Holland et al. (2021) [121]	United Kingdom	Survey: 5,063 dog owners<br><br>Diary: 64 general public, 262 Generation Pup project participants	Survey: 86% female, 14% male, less than 1% preferred not to say or identified as 'other'<br><br>Age group most represented was 55-64 (25.2%)<br><br>Diary: 57% female; 6 male; 1 did not disclose<br><br>Age group most represented was 55-64 (31.3% of general public respondents and 29.4% of Generation Pup	Dogs	Open-ended survey questions and an electronic diary	-	Survey: What they were enjoying about dog ownership at the current time; what they were concerned about regarding their dog(s) at the current time<br><br>Diary: how dog's life and their relationship with their dog was impacted by lockdown	Survey: May 2020<br><br>Diary: March - May 2020

			project respondents)					
Jezierski et al. (2021a) [122]	Authors in Poland, United Kingdom and United States of America; participants were international	324 cat owners	87.6% female, 11.7% male<br><br>Mean age 39	Cats	Cross-sectional survey	-	Changes in cat's care, behavior and health during pandemic; difficulties and/or advantages of having a cat during the pandemic	May - June 2020
Jezierski et al. (2021b) [123]	Authors in Poland, United Kingdom and United States of America; participants were international	688 dog owners	88.2% female, 11.8% male<br><br>Mean age 43	Dogs	Cross-sectional survey	-	Changes in dog care, behavior and health during pandemic; difficulties and/or advantages of having a dog during the pandemic	May – June 2020
Johnson & Volsche (2021) [124]	United States of America	234 adults with at least one companion animal	82.9% cisgender female, 11.5% cisgender male, 2.1% gender non-conforming, 1.3% who identified as queer, 0.9% transgender female, 0.9% non-binary, 0.4% did not answer<br><br>Age group most	Not reported	Cross-sectional survey	-	Changes in relationship with pet during shelter-in-place; role of animals in the home; attachment to pets	Not reported

			represented was 25-35 (30.3%)					
Junça-Silva et al. (2022) [125]	Portugal	320 teleworkers with pets, 81 teleworkers without pets	Non-pet owners: 51% male<br><br>Mean age 32.09<br><br>Pet owners: 62% female<br><br>Mean age 31.87	Dogs	Cross-sectional survey	Quality of telework experience (effectiveness/ productivity, organizational trust, interference between home and work life, flexibility); positive affect; self-rated job performance	Emotional attachment to pets; physical closeness to pets	February - March 2021
Kirnan et al. (2022) [126]	United States of America	4 college students with emotional support animals	75% female, 25% male	3 participants had cats, 1 had a bearded dragon	Qualitative interviews	General feelings during COVID-19	-	Not reported
Kogan et al. (2021a) [127]	Authors in United States of America; participants were international	5,061 dog or cat owners	89% female, 10% male, 1% non-binary<br><br>Age group most represented was 40-59 (43%)	Dogs, cats	Cross-sectional survey	-	Impact of pets on COVID-related feelings; bond with pet	March - May 2020
Kogan et al. (2021b) [128]	Authors in the United States of America; participants were international	4,105 dog owners	88.3% female, 10.8% male, 0.8% non-binary, 0.1% did not answer	Dogs	Cross-sectional survey	Social support; frustration	Changes in time spent with dog; changes in bond with dog; level of bond with dog; changes in dog behaviors;	March – May 2020

							veterinary care; dog-related concerns; psychological impact of having dogs during the pandemic	
Kogan et al. (2021c) [129]	Authors in the United States of America; participants were international	956 cat owners	91.9% female, 7.8% male, 0.3% non-binary	Cats	Cross-sectional survey	Social support; frustration	Changes in time spent with cat; changes in bond with cat; veterinary care; other cat-related concerns; changes in behavior; psychological impact of having cats during the pandemic	March – May 2020
Koochaknejad et al. (2022) [130]	Authors in Iran; participants were international	168 home aquarium owners	56.5% male, 38.7% female, 4.8% non-binary<br><br>44.6% aged 15-24, 55.4% aged 25-64	Fish	Cross-sectional survey	-	Belief that aquarium had stress-relieving benefits during pandemic; thoughts about giving up non-fish pets due to perceiving them to be at risk of coronaviruses; money spent on aquariums; welfare of fish during pandemic	March – April 2021
Krouzecky et al. (2022) [131]	Authors in Austria; unclear where	242 animal caregivers	83.8% female, 15.4% male, 0.8% other	Various	Cross-sectional survey	Depressive symptoms; quality of life; loneliness; social	Emotional attachment to companion animal; opinion of	Not reported

	participants were from		Mean age 36			support; pandemic-related stressors/concerns	the animals’ role during the pandemic and in the management of Long-Covid	
Kuehne et al. (2022) [132]	Authors in United Kingdom; participants from Germany (33%), United Kingdom (29%), Hong Kong (18%), Ireland (8%), other (12%)	49 adults	31% male, 69% female<br><br>Mean age 36.5 (range 23-61)	Dogs, cats, others	Cross-sectional survey	General wellbeing; social connectedness and social assurance; coronavirus anxiety	Pet attachment	Not reported
Lau & Oliva (2023) [133]	Australia	534 adults living alone	Gender and age reported for various sub-groups but not the overall sample	Dogs, cats	Cross-sectional survey	Loneliness; COVID-19 experiences; mindfulness; mindfulness or meditation experiences, both during lockdown and in general	Pet ownership – dog / cat / none; dog-walking frequency	August – September 2020
Law et al. (2022) [134]	New Zealand	261 adults	7% male, 93% female<br><br>Mean age 43.87	Not reported	Longitudinal surveys	Anxiety; depression; positive mood; perceived risk of getting COVID-19; worry about contracting COVID-19	Pet owner – yes/no	T1: May 2020<br>T2: August – September 2020<br>T3: March – April 2021
Lee et al. (2022) [135]	Korea	249 dog owners	83.1% female, 16.9% male	Dogs	Cross-sectional survey		Attachment to dogs; perceived outcomes of dog walking	Fall 2021

			Age group most represented was 29 or under (43.4%)					
Lima et al. (2022) [136]	Portugal	509 adults (345 dog owners, 164 non-dog owners)	Dog owners: 79% female, 21% male<br><br>Mean age 38<br><br>People without dogs: 69% female, 31% male<br><br>Mean age 40	Dogs	Cross-sectional survey	COVID-19 quarantine; social support; anxiety; depression	Lived with a dog – yes/no; lived with any other companion animal – yes/no; if dogs, dog-owner relationship was assessed, including emotional closeness and perceived costs; dog owners also reported time spent walking and whether they had considered relinquishing their pet	May – June 2020
Lukoševičiūtė & Šmigelskas (2022) [137]	Lithuania	19 adolescents	52.6% female, 47.4% male<br><br>Mean age 13.8, range 11-17	Not reported	Qualitative interviews	Mental health during pandemic; hardest part of pandemic; what helped to overcome difficulties; new discoveries the pandemic has brought	-	April - July 2021
Martin et al. (2021) [138]	United States of America	768 dog owners, 767 potential dog owners	Dog owners: 58.2% female, 41.67% male,	Dogs	Cross-sectional survey	Perceived social support; depression;	Attitude towards pets; commitment to pets	November 2020; additional 350 dog owners

		(interested in having a dog in future)	0.13% prefer not to say<br><br>Mean age 45.43, range 18-82<br><br>Potential dog owners: 58.28% female, 41.59% male, 0.13% other<br><br>Mean age 42.41, range 18-99			anxiety; happiness		and 350 potential dog owners recruited February 2021
Martinez-Caja et al. (2022a) [139]	Authors in Belgium and the Netherlands; participants were international	6,520 adults (83.7% pet owners)	86.7% female, 13.1% male, 0.2% other<br><br>Age group most represented was 26-35 (24.4%)	Dogs, cats, horses, rabbits, birds, fish, small rodents, other	Cross-sectional survey	Loneliness; positive and negative affect	Pet ownership – yes/no; pet owners reported how many animals they had, species of animals, and completed a measure of human-animal bond	April – May 2020
Martinez-Caja et al. (2022b) [140]	Authors in Belgium and the Netherlands; participants were international	5,454 pet owners	10.14% male, 89.38% female, 0.48% other<br><br>Age group most represented was 26-35 (24.27%)	Dogs, cats, horses, rabbits, birds, small rodents, fish, reptiles, other (e.g. goats, sheep, alpacas, tarantulas, ferrets)	Cross-sectional survey	-	Type of animal; relationship with pets; changes in pets' routine; time spent with pets and enjoyment of time with pets; behavioral changes in pets	April – May 2020

Matijczak et al. (2021) [141]	United States of America	1,453 sexual and gender minority individuals who lived with a companion animal	89.7% cisgender female, 7% cisgender male, 1.1% genderqueer / gender non-conforming, 0.2% transgender male, 0.1% prefer to self-describe, 1.7% multiple identities, 0.2% prefer not to say<br><br>Age not reported	Dogs, cats, fish, reptiles, birds, small mammals, horses, other (e.g. cow, pig, donkey, chicken, axolotl)	Cross-sectional survey	-	Whether concerns about pet's welfare would lead to delaying or avoiding COVID-19 testing or treatment; attachment to pets	April – July 2020
McDonald et al. (2021) [142]	United States of America	1,942 adults with at least one companion animal	89.8% cisgender female, 7.0% cisgender male, 1.1% genderqueer/ gender non-conforming, 0.1% transgender female, 0.5% transgender male, 1.4% multiple identities, 0.3% missing data	Various	Cross-sectional survey	Psychological adjustment (anxiety, depression, hostility, interpersonal sensitivity, obsessive-compulsive, phobic anxiety, somatization)	Attachment to pets	April - July 2020

			Mean age 39.68, range 18-85					
Moore et al. (2020) [143]	Canada	1,472 parents of children or youth	47% female<br><br>Mean age 45.12	Dogs	Cross-sectional survey	Child movement behaviors; change in child movement and play behaviors; parental support for child's movement and play behaviors	Dog ownership – yes/no	Approximately 1 month after COVID was declared a global pandemic by the WHO
Morgan et al. (2020) [144]	Israel	2,906 dog owners	Not reported	Dogs	Cross-sectional survey	Effect of pandemic on stress; effect of pandemic on financial wellbeing; concerns about own health	Wellbeing of dog; behavior of dog during lockdown; frequency of dog-walking	March – April 2020
Morris et al. (2021) [145]	Canada	12 people with companion animals, who met the low-income threshold to access support for veterinary fees from the Vancouver Humane Society	Not reported	Not reported	Qualitative interviews	-	COVID-related challenges in accessing veterinary care; resources and support to address these challenges	December 2020 – May 2021
Mueller et al. (2021) [146]	United States of America	1,033 early adolescents (T1)<br>357 early adolescents (T2)	T1: 50% female, 49% male, 1% other<br><br>Mean age 12.69	Dogs, cats, reptiles, fish, horses, other	Follow-up study	Loneliness; coping with stress	Pet in the family – yes/no; average daily time spent with pet; pet attachment	T1: October - December 2019; T2: June - July 2020

			T2: 65% female, 34% male, 1% other<br><br>Mean age: 13.02					
Mueller et al. (2022) [147]	United States of America	6,069 adolescents	49.5% female, 50.5% male<br><br>Mean age 12.57	Dogs, cats, horses, fish, small animals, other	Cross-sectional survey	Positive affect; perceived stress; healthy coping behaviors; walking routine; regular outdoor time	Pet in the family – yes/no	June 2020
Muzzatti & Grieve (2022) [148]	Canada and United States of America	Not reported	Not reported	Not reported	Mixed-methods: surveys, interviews, discussions among veterinary technicians and technologists	Not reported	Not reported	Not reported
Namekata & Yamamoto (2021) [149]	Japan	180 university students	68.9% female, 31.1% male<br><br>Mean age 19.4, range 18-23	Dogs, cats, small animals, other	Cross-sectional survey	Perceived difficulties of the pandemic; ways of relieving stress; mood states	Companion animal ownership; type of pet; attachment to companion animal	June - July 2020
O'Connor et al. (2022) [150]	United States (unclear if some participants were international)	284 people who had lost a pet during the COVID-19 pandemic	206 females, 31 males, 7 non-binary<br><br>Age group most represented was 18-30 (81.2%)	Dogs, cats, reptiles, fish, birds, rodents, pigs, exotic animals, other	Cross-sectional survey	Other losses during the pandemic; isolation; social support; stress; grief	Attachment to pets; continuing bonds to pets	February – June 2021

Ogata et al. (2023) [151]	United States of America	4,237 adults	Non-pet owners: 269 female, 381 male, <5 non-binary<br>Dog owners: 850 female, 888 male, 12 non-binary<br>Cat owners: 907 female, 845 male, 18 non-binary<br><br>Mean age at baseline: 37 (range 19-71) for non-pet owners, 37 (range 18-79 for dog owners), 36 (range 18-72) for cat owners	Dogs, cats	Longitudinal	Perceived stress; perceived loneliness	Characteristics of pet; length of ownership; whether respondent was primary caregiver; other household pets; owner-pet relationship (i.e., perceived closeness)	Cohort established June 2020; pre-pandemic (February 2020) and lockdown (April 2020) data collected retrospectively; follow-up surveys in September 2020, January 2021, April 2021, August 2021, December 2021
Oliva & Green (2021) [152]	Australia	67 dog owners	88% female, 12% male<br><br>Age not reported	Dogs	Intervention trial: six-week Dog Assisted Mindfulness (DAM) involving guided imagery vs. Dog Interactions (DI) involving different play activities with dogs vs. control group	Mindfulness; loneliness; perceptions of interventions (for intervention groups only)	Perceived emotional closeness to dogs	August – October 2020

Oliva & Johnston (2021) [153]	Australia	384 people who lived with no other humans (30.2% lived with a dog, 32.81% lived with a cat)	14.1% male, 85.4% female female, 0.3% non-binary, 0.3% unspecified<br><br>Mean age 50.92 (range 23 – 89)	Dogs, cats	Cross-sectional survey	Mindfulness and meditation experience; loneliness; depression, anxiety and stress	How being a pet owner impacted their experience of COVID-19; how the pandemic was perceived to have affected pets; pet interaction	May 2020
Oliver-Hall et al. (2021) [154]	United Kingdom	352 (189 people with multiple sclerosis, 163 controls)	People with multiple sclerosis: 23% female, 76% male, 1% not answered<br><br>Mean age 54<br><br>Controls: 47% female, 50% male, 2% not answered<br><br>Mean age 53	Dogs, cats, other	Cross-sectional survey	Satisfaction with (participation in) social roles; quality of life; self-efficacy	Pet ownership – yes/no; type of pet; number of pets; attachment to pets; perceived role of pets during pandemic; pet-related causes of concern	Not reported
Oomen et al. (2021) [155]	Belgium, Netherlands and United Kingdom	1,044 (613 with a diagnosis of autism)	68.8% female, 29.7% male, 16 people – other<br><br>Mean age, autism group: 38.36, range 18-78<br><br>Mean age, non-autism group: 38.38, range 18-81	Not reported	Cross-sectional survey	COVID-19 experiences; depression and anxiety; worries; social life and routines; mental health needs during pandemic; which changes in everyday life were most difficult, most stressful, and most pleasant	Living with pets – yes/no	April - May 2020

Owczarczak-Garstecka et al. (2021) [156]	United Kingdom	584 dog owners	88.7% female, 10.5% male, 0.9% not disclosed<br><br>Age group most represented was 30-50 (43.2%)	Dogs	Cross-sectional survey	-	Number of dogs in household; their size, age and sex; dog's perceived energy levels; relationship with dog; frequency od dog's interactions with people and dogs pre-lockdown; dog's recall reliability; frequency and duration of dog walking during lockdown and before; perceived changes in dogs' interactions; who walks dog; changes in human's walking habits; changes in location of dog walks; perceptions of dog ownership	May - June 2020
Packer et al. (2021) [157]	United Kingdom	Owners of 1,148 puppies purchased in 2019 and owners of 4,369 puppies purchased during the pandemic	2019: 92% female<br>2020: 90% female<br><br>Age group most represented was 45-54	Dogs	Comparison study using surveys	-	Motivations for purchasing a puppy; pre-purchase behaviors; purchase behaviors; COVID-specific impacts	November – December 2020

			(25.2% in 2019, 24.6% in 2020)					
Packer et al. (2023) [158]	United Kingdom	1,148 ‘2019 puppies’, 4,369 ‘2020 pandemic puppies’, 2,080 ‘2021 puppies’	94.8% female (owners of 2021 puppies), 90% female (owners of 2020 puppies), 92% female (owners of 2019 puppies)<br><br>Age group most represented was 45-54 (24.1%)	Dogs	Comparison study using surveys	-	Motivations for purchasing a puppy; pre-purchase behaviors; purchase behaviors; COVID-specific impacts	February – April 2022
Pawar et al. (2021) [159]	India	100 people with companion animals	Not reported	Dogs, cats	Cross-sectional survey	-	Behavioral changes in pets; difficulties fulfilling pets‘ needs in lockdown	Not reported
PDSA (2020) [160]	United Kingdom	February 2020 survey: 4,767 pet owners<br><br>August 2020 survey: 4,103 pet owners	Not reported	Dogs, cats, rabbits	Cross-sectional survey (with some comparisons to other time-points)	-	Animal wellbeing and behavior; impact of pandemic on animals	February 2020, compared to data from August 2020 and annual surveys dating back to 2011 for trend analysis
PDSA (2021) [161]	United Kingdom	4,579 pet owners	Not reported	Dogs, cats, rabbits	Cross-sectional survey	-	Animal wellbeing and behavior; impact of pandemic on animals	May 2021

Phillipou et al. (2021) [162]	Australia	263 (138 pet owners vs. 125 non-owners)	Pet owners: 88% female<br><br>Mean age 25.85<br><br>Non-owners: 80% female Mean age 26.45	Cats, dogs	Cross-sectional survey	Loneliness; resilience; quality of life; negative emotional states	Ownership of a cat or dog – yes/no	September 2020
Piotti et al. (2021) [163]	Authors in Italy, Greece and UK; participants were international	235 pet owners	85% female, 14% male, 1% other/prefer not to say<br><br>Age group most represented was 35-44 (30%)	Dogs, cats	Cross-sectional survey	Perceived risk of COVID-19; social interactions; financial effects of the pandemic; wellbeing	Pet quality of life; pet-owner relationship	May – July 2020
Platto et al. (2022) [164]	China	261 dog or cat owners	Not reported	Dogs, cats	Cross-sectional survey	-	Type of pet owned (dog / cat / both); difficulties finding pet food and veterinary care during lockdown; management of pets; behavioral issues; companion animal bond; stress-related behaviors	July 2020 – June 2021
Ratschen et al. (2020) [165]	United Kingdom	5,926 adults	78.6% female, 20.6% male, 0.6% other, 0.2% prefer not to say	Dogs, cats, small mammals, birds, fish, reptiles or amphibians, horses or	Cross-sectional retrospective survey	Mental wellbeing; mental health; loneliness	Animals in the household – yes/no; how many and which species; comfort from companion animals (Comfort	April - June 2020; participants provided current data and also retrospectively

			Age group most represented was 45-54 (23.8%)	ponies, farm animals, other			from Companion Animals Scale); role of companion animals during lockdown	provided 'before lockdown' data
Reniers et al. (2022) [166]	Netherlands	9 (5 older adults receiving long-term care at home, 4 family caregivers of people with dementia)	55% female, 45% male<br><br>Mean age 72.1, range 62-82	Dogs, cats	Qualitative interviews	Experiences and quality of life during the pandemic	Bond with companion animals; change in significance of pets; benefits and challenges of having a pet during lockdown	May - September 2021
Ribeiro et al. (2023) [167]	Brazil	300 dog owners	52% female, 48% male<br><br>Age range 25 – 44	Dogs	Cross-sectional survey	Quality of life	Environment where animal lived; changes in routine; behaviors; physical health; quality of life	July – September 2020
Riggio et al. (2022) [168]	Italy	548 cat owners	81.6% female<br><br>Age group most represented was 36-45 (30.7%)	Cats	Cross-sectional survey	Effects of confinement on emotions, finances, health and quality of life	How pet-owner relationship had been affected by the pandemic; how pet's quality of life was affected by pandemic; whether they became angry with their pet more frequently; behavioral changes in pet	April 2020
Rombach & Dean (2021) [169]	United States of America	206 pet owners	156 males, 50 females	Cats, dogs	Cross-sectional survey	-	Pet care and engagement; experiences with pet food	July 2021

			Age group most represented was 25-34 (63.1%)				insecurity and anxiety; shopping for and feeding pets during the pandemic	
Sacchettino et al. (2023) [170]	Italy	310 dog owners	80.08% female, 19.92% male<br><br>Age group most represented was 30-40 (30.15%)	Dogs	Comparison study of dogs who were puppies in lockdown vs. dogs born after lockdown	-	Dog personality and behavior	February – September 2022
Sánchez-Ferrer et al. (2022) [171]	Spain	3,890 parents reporting on children	Children: 51.4% male<br><br>Mean age 6.78	Not reported	Cross-sectional survey	Child's acceptance of and adaptation to the pandemic; perception of whether child might have trouble returning to normal activities; sleep problems; appetite changes; screen time; emotional state during pandemic	Pets in the home – yes/no	April 2020
Scholtz (2022) [172]	Author in South Africa; participants international	77 remote workers	90% female, 9% male, 1% missing data<br><br>Mean age 43	Dogs, cats, other (but all participants who chose 'other' also had dogs or cats)	Cross-sectional survey with qualitative descriptive responses	-	'Do you have anything to add regarding your experience as a pet owner and remote worker during COVID-19?'	January - April 2022

Shah et al. (2021) [173]	Authors in Pakistan; participants international	678 individuals	57.2% female, 42.8% male<br><br>Age groups most represented were 18-24 and 25-34 (% not reported)	Not reported	Cross-sectional survey	Depression, anxiety and stress	'Impact of pets'	Not reported – 'during quarantine / lockdown'
Sherlock et al. (2023) [174]	Ireland	662 dog owners	79% female, 20% male, 1% non-binary<br><br>Age group most represented was 18-24 (36%)	Dogs	Cross-sectional survey	-	Dog's lifestyle and care, including diet, walking, parasite control practices, veterinary care	January 2022
Sherwell et al. (2023) [175]	Authors in United Kingdom and United States of America; unclear if participants were international	1,106 dog owners (at baseline)	Not reported	Dogs	Longitudinal	-	Dog behaviours; changes in management of dogs	Recruitment took place May – July 2021; surveys were completed each month for up to 8 months
Shoesmith et al. (2021a) [176]	United Kingdom	5,323 adults	79.3% female, 20% male, 0.5% other, 0.2% prefer not to say<br><br>Age group most represented was 45-54 (24.8%)	Dogs, cats, small mammals, birds, fish, reptiles or amphibians, horses or ponies, farm animals, other	Cross-sectional retrospective survey with open-ended questions	Mental health	Do you have animals that live with or near you that you or anyone in your household are the main caretaker for – yes/no; how many animals; which species; changes in	April – June 2020

							animals' welfare and behavior; human-animal bond and interactions	
Shoesmith et al. (2021b) [177]	United Kingdom	934 companion animal owners	78.3% female, 20.7% male, 0.9% other, 0.1% prefer not to say<br><br>Age group most represented was 55-64 (27.3%)	Dogs, cats, small mammals, fish, horses or ponies, birds, reptiles, farm animals, amphibians	Cross-sectional survey with open-ended questions	-	Open-ended question asking about experiences with animals during COVID-19	April – June 2020
Shoesmith et al. (2021c) [178]	United Kingdom	249 people with severe mental illness	46.6% cisgender female, 51.4% cisgender male, 2% transgender<br><br>Age group most represented was 45-54 (55%)	Dogs, cats, small mammals, fish, birds, reptiles or amphibians, farm animals, other	Cross-sectional survey including an open-ended question	General mental health; general physical health; global health and wellbeing; loneliness	Animals living with or near you which you or someone in the household are the main caretaker of – yes/no; species; engagement with companion animals if yes; free-text question asking for any other comments about what animals meant to participants during the pandemic	January – March 2021
Tan et al. (2021) [179]	Singapore	534 (431 pet owners, 103 non-owners)	Pet owners: 89.3% female<br><br>Median age 33	Dogs, cats, small mammals or birds	Cross-sectional survey	Physical activity levels; general health, emotional wellbeing, energy	Sharing a house with a dog, cat, small mammal or bird – yes/no;	May – July 2020

			Non-owners: 79.6% female<br><br>Median age 29			and social functioning	attachment to pets; main caregiver or not; level of involvement with pet care	
**van der Velpen et al.** (2022) [180]	Netherlands	5,017 older adults	56.9% female<br><br>Mean age 68.7	Not reported	Prospective longitudinal cohort study	Mental and social health; healthcare utilization; loneliness; social isolation; social connectedness	Pet ownership – yes/no	T1: April 2020; questionnaires completed at three time-points between April – July 2020
Victor & Mayer (2023) [181]	South Africa	10 pet owners	100% female<br><br>Mean age 25.3 (range 22 – 29)	Dogs (n=9), cat (n=1)	Qualitative interviews	Not reported	Not reported	Not reported
Vučinić et al. (2022) [182]	Serbia	216 dog owners	74% female, 26% male<br><br>85% aged 18-44, 15% aged 45-64	Dogs	Cross-sectional survey	-	Were dogs walked for at least 150 min/week before and during the state of emergency	March – April 2020
Wan et al. (2022) [183]	United States of America	187 employees with companion animals	50% male, 50% female<br><br>Mean age 36.66	Not reported	Follow-up study with a 1-week time-lag	Job insecurity; stress; substance use; emotional exhaustion; depression	Pet attachment support	April 2020
Ward et al. (2021) [184]	United Kingdom (22 participants in Scotland, 2 in England)	24 (5 veterinarians, 6 horse owners with horses at home, 5 horse owners with horses at an external establishment,	Not reported	Horses	Qualitative interviews	Challenges in the management of horses during the pandemic; conversations often drifted towards owners' mental health and wellbeing	-	May - July 2020

		4 farriers, 4 equine welfare centre managers						
Wells et al. (2022) [185]	United Kingdom	249 adults (146 companion animal owners, 103 non-owners)	78.3% female, 21.7% male<br><br>Age group most represented was 18-29 (53%)	Dogs, cats	Cross-sectional survey	Depression; positive experiences; loneliness; stress	Ownership of cat or dog – yes/no; Attachment to pets	January 2021
Williams et al. (2020) [186]	United Kingdom	6,259 horse owners	Not reported	Horses	Cross-sectional survey which included an open-ended question	-	Impact of pandemic on horse management; impact of pandemic on interactions with horse; whether financial implications of pandemic were predicted to affect horse care	March – April 2020
Woolley et al. (2022) [187]	United Kingdom	1,265 (COVID-19 lifestyle surveys, compared to 12,451 surveys from previous years); illness data from 16,115 surveys from 4,110 dog owners	Not reported	Dogs	Longitudinal	-	Exercise quantity; feeding; insurance status; sleeping location; baths; anti-parasite treatment; illnesses; veterinary care	Data from March – July 2020 compared to data from the same dates in previous years from 2011 to 2019
Wriedt (2022) [188]	Author in United States;	24 people who acquired a dog	Not reported	Dogs	Cross-sectional	How COVID impacted life	Factors influencing	September 2021

	data on participant location not collected	between February – December 2020			survey with open-ended questions		decision to get a dog; experiences of having a new dog during the pandemic	
Wu et al. (2021) [189]	Canada	12 people with companion animals, including 8 with (dis)abilities	92% female, 8% male<br><br>9 aged 40-59, 3 aged 18-39	Not reported	Qualitative interviews	COVID-19 challenges (including those relating to animals); resources and support to address these challenges	Suggestions for veterinary services and animal protection agencies; lessons learned for animal caregivers in future pandemics	December 2020 – May 2021
Wu et al. (2023) [190]	Canada	12 people who cared for animals and sought veterinary treatment	92% female, 8% male<br><br>63% aged between 40-59, 27% aged between 18-39	Not reported	Qualitative interviews	-	Experiences obtaining veterinary care during the pandemic, including challenges, coping strategies, recommendations	December 2020 – May 2021
Xin et al. (2021) [191]	Authors in China; 99.6% of participants in China, 0.4% international	756 people; a second survey was launched with 68 participants	27.6% male, 72.4% female<br><br>Age group most represented was 19-35 (61.6%)	Dogs, cats, rabbits, turtles, birds, other	Cross-sectional survey	Insomnia; hypersomnia; anxiety; depression; fatigue; lack of enthusiasm; inattention; uncertainty; concerns about infection	Pet ownership – yes/no; type of pet; period of ownership; purpose of having pets; relationships with pets; attitudes toward pets; change in interaction with pets during pandemic	April 2020
Zablan et al. (2023) [192]	Australia	177 older adult	89.8% female	Dogs, cats, small mammals,	Cross-sectional semi-	Experience of the pandemic	Companion animal owner status; primary	November 2020 – January 2021

		companion animal owners	Mean age 70.72 (range 65 – 84)	fish, birds, horses, livestock	structured survey		animal carer status; type(s) of animals owned; relationship with pet during pandemic and how it changed over time; perceived impact of how pets influenced wellbeing and how pandemic influenced pet care	
Zainel et al. (2023) [193]	Qatar	6,608 children and adolescents	50.8% male, 49.2% female<br><br>62.8% aged between 7-12, 37.2% aged between 13-18	Not reported	Cross-sectional survey with open-ended question	Open-ended question about what made people happy during lockdown	-	June – July 2020
Zaninotto et al. (2022) [194]	United Kingdom & Italy	96 individuals	Italy: 45% male (mean age 40), 55% female (mean age 41)<br><br>UK: 20% male (mean age 43), 80% female (mean age 23)	Dogs	Longitudinal	Pandemic impact as a traumatic personal event; mindfulness; daily routines and habits; short version of SCL90-R; depression, anxiety and stress; sleep disorders; alcohol and nicotine use; cognitive tasks assessing attention and vigilance	Dog walking – yes/no	Data collected once a week for four weeks 'during first-wave lockdowns'
